# Arginine dependency is a therapeutically exploitable vulnerability in chronic myeloid leukaemic stem cells

**DOI:** 10.15252/embr.202256279

**Published:** 2023-07-25

**Authors:** Kevin M Rattigan, Martha M Zarou, Zuzana Brabcova, Bodhayan Prasad, Désirée Zerbst, Daniele Sarnello, Eric R Kalkman, Angela Ianniciello, Mary T Scott, Karen Dunn, Engy Shokry, David Sumpton, Mhairi Copland, Saverio Tardito, Johan Vande Voorde, Francis Mussai, Paul Cheng, G Vignir Helgason

**Affiliations:** ^1^ Wolfson Wohl Cancer Research Centre, School of Cancer Sciences University of Glasgow Glasgow UK; ^2^ Paul O'Gorman Leukaemia Research Centre, School of Cancer Sciences University of Glasgow Glasgow UK; ^3^ Cancer Research UK Beatson Institute Glasgow UK; ^4^ Institute of Immunology and Immunotherapy University of Birmingham Birmingham UK; ^5^ Bio‐cancer Treatment International Ltd, Hong Kong Science Park Shatin New Territories Hong Kong

**Keywords:** amino acids, leukaemic stem cells, metabolism, therapy resistance, Cancer, Immunology, Metabolism

## Abstract

To fuel accelerated proliferation, leukaemic cells undergo metabolic deregulation, which can result in specific nutrient dependencies. Here, we perform an amino acid drop‐out screen and apply pre‐clinical models of chronic phase chronic myeloid leukaemia (CML) to identify arginine as a nutrient essential for primary human CML cells. Analysis of the Microarray Innovations in Leukaemia (MILE) dataset uncovers reduced ASS1 levels in CML compared to most other leukaemia types. Stable isotope tracing reveals repressed activity of all urea cycle enzymes in patient‐derived CML CD34^+^ cells, rendering them arginine auxotrophic. Thus, arginine deprivation completely blocks proliferation of CML CD34^+^ cells and induces significantly higher levels of apoptosis when compared to arginine‐deprived cell lines. Similarly, primary CML cells, but not normal CD34^+^ samples, are particularly sensitive to treatment with the arginine‐depleting enzyme, BCT‐100, which induces apoptosis and reduces clonogenicity. Moreover, BCT‐100 is highly efficacious in a patient‐derived xenograft model, causing > 90% reduction in the number of human leukaemic stem cells (LSCs). These findings indicate arginine depletion to be a promising and novel strategy to eradicate therapy resistant LSCs.

## Graphical abstract

A complete lack of urea cycle function is a specific feature of primary CML patient samples. This makes them sensitive to arginine deprivation *in vitro* and *in vivo*.

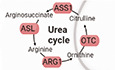

## Introduction

Multiple metabolic pathways have been found to be deregulated in leukaemia, including branched chained amino acid and glutamine metabolism (Škrtić *et al*, 2011; Farge *et al*, 2017; Kuntz *et al*, 2017; Raffel *et al*, 2017; Gallipoli *et al*, 2018). Further metabolic vulnerabilities emerge due to the inability of leukaemic cells to synthesise nonessential amino acids, which can be exploited by clinical, long‐lasting enzymes that reduce circulating levels of specific amino acids (Mussai *et al*, 2015; Cramer *et al*, 2017). In this context, arginine has emerged as a promising candidate with arginine‐degrading enzymes being tested in clinical trials against multiple types of leukaemia, liver cancer, melanoma, prostate cancer, lymphoma, glioblastoma, mesothelioma, sarcoma, and lung cancer. However, many of these trials have not been successful with upregulation of arginine recycling enzymes, such as argininosuccinate synthase 1 (ASS1), being reported as an escape mechanism (Zou *et al*, 2019).

Chronic myeloid leukaemia (CML) is a myeloproliferative disease, initiated by a reciprocal translocation between chromosomes 9 and 22 t(9;22)(q34;q11) leading to the formation of the Philadelphia chromosome, containing constitutively active BCR‐ABL1 oncogenic fusion‐protein (Rowley, 1973; Groffen *et al*, 1984; Konopka *et al*, 1984). The natural history of CML is that most patients present in chronic phase before inexorably progressing to the more aggressive accelerated phase or lethal blast phase if left untreated (Giralt *et al*, 1995). As chronic phase CML lacks the genetic complexity associated with other types of leukaemia and has a well‐defined leukaemic stem cell (LSC) population, CML is an ideal model to explore responses to targeted therapeutics. While the introduction of tyrosine kinase inhibitors (TKIs) such as imatinib has increased survival of patients in chronic phase (Druker *et al*, 2006), the failure of TKIs to eradicate LSCs that can re‐establish disease (Corbin *et al*, 2011; Hamilton *et al*, 2012) means that the majority require life‐long TKI treatment, which is associated with significant morbidities and toxicities, and treatment discontinuation is frequently unsuccessful (Rousselot *et al*, 2006; Steegmann *et al*, 2016).

The ability of acute myeloid leukaemia (AML) cell lines to rapidly upregulate ASS1 compared to T‐cells has been reported to be due to AML having more accessible chromatin (Crump *et al*, 2021). However, this study compared AML cell lines to primary T‐cells, so whether this is the case in primary patient samples remains to be determined. Our previous studies on arginine dependency in leukaemic blast cells showed pronounced effect, although these experiments were done in media without urea cycle intermediates, thereby preventing potential rescue via the urea cycle. Whether therapy‐resistant LSCs are auxotrophic for non‐essential amino acids in the presence of their circulatory precursors is unknown. Here, we conducted a systematic amino‐acid dropout screen and identified arginine as essential for proliferation and viability of primary chronic phase CML cells. We further investigated the effect of arginine deprivation, alone and in combination with imatinib, against human CML LSCs *in vitro* and *in vivo*.

## Results and Discussion

### Physiological levels of urea cycle intermediates fail to rescue leukaemic arginine dependency

**Figure 1:**
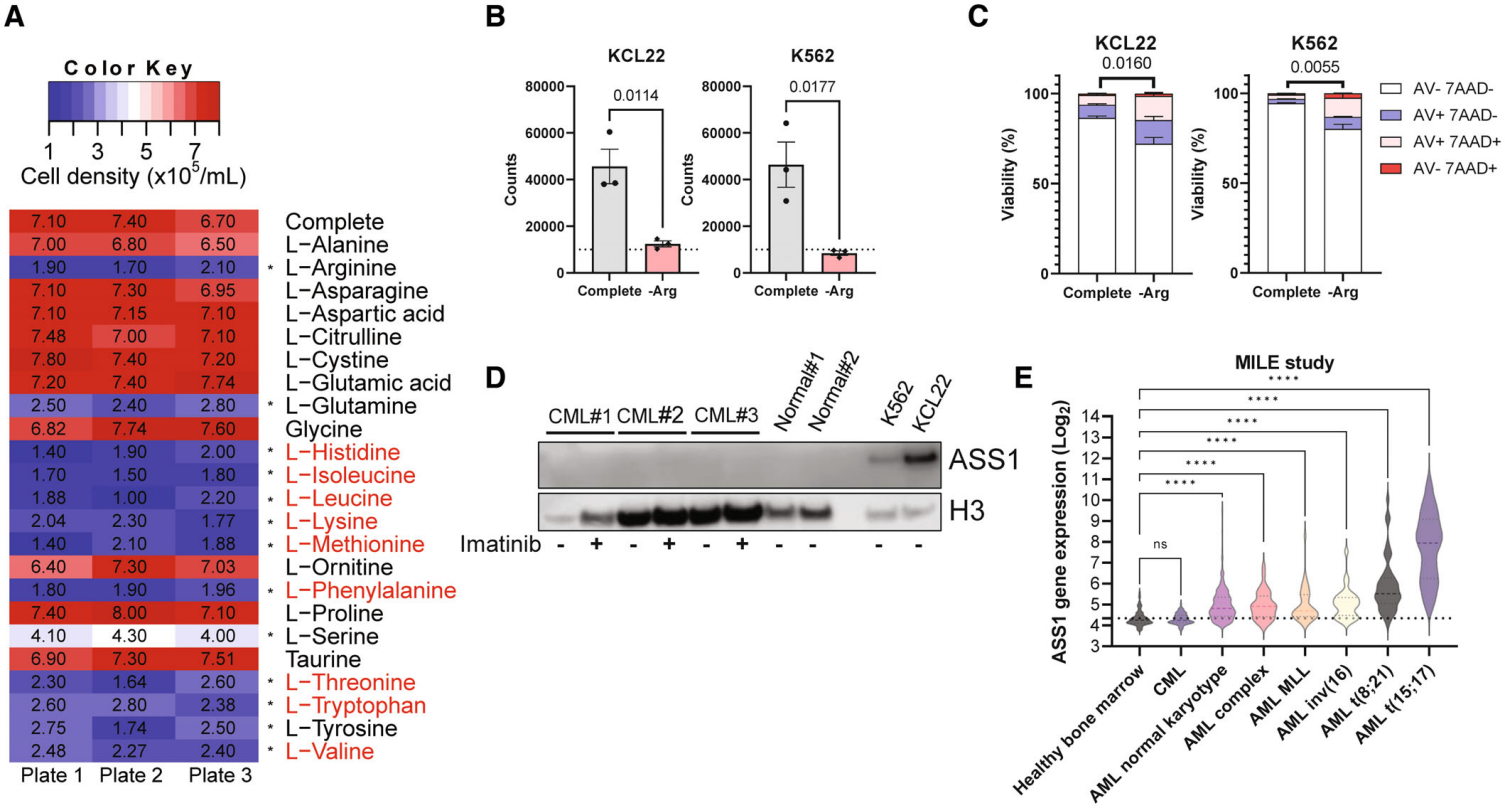
Physiological drop‐out screen reveals arginine dependency in CML Cell number (per well) from K562 cell line grown for 72 h in complete medium or medium deficient in indicated amino acid. Cells were seeded at 10,000 cells (dashed line) in 200 μl/well in three replicate plates. Essential amino acids are in red.Indicated cell lines grown for 72 h in complete medium or medium deficient in arginine and cell density recorded (×10^5^/ml). Three independent experiments are shown with mean and SEM. The dotted line shows seeding density (10,000 cells/100 μl).Indicated cell lines grown for 72 h in complete medium or medium deficient in arginine and viability measured. Three independent experiments are shown with mean and SEM. The live cell fractions (Annexin V−, 7‐AAD−) were used for statistical analysis.Western blotting was used to visualise ASS1 protein levels in untreated or imatinib treated (2 μM, 48 h) CML CD34^+^ samples, normal CD34^+^ samples and indicated cell lines following 16 h arginine starvation.ASS1 expression from the MILE study. The dotted line revers for average of Healthy Bone Marrow. Data information: For statistical analysis, an ordinary one‐way ANOVA with Dunnett's correction for multiple comparisons was performed for A with * referring to *P* < 0.0001, unpaired *t*‐tests were used for (B, C), a Kruskal‐Wallis test was performed on (E) with **** referring to *P* < 0.0001 in (E). Source data are available online for this figure.

**Figure EV1:**
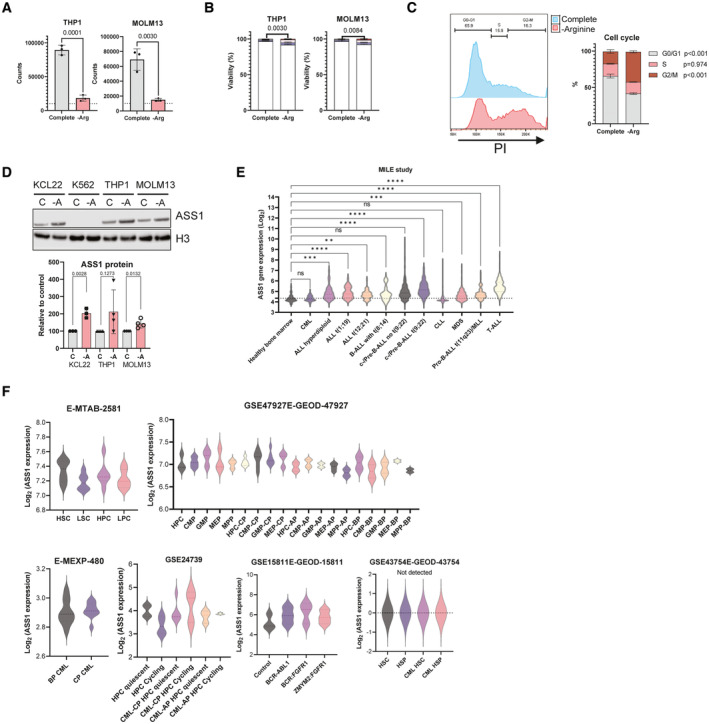
Absence of ASS1 is a feature of CML patient samples Indicated cell lines grown for 72 h in complete medium or medium deficient in arginine and cell number recorded (per well). Cells were seeded at 10,000 cells (dashed line) in 200 μl/well in three replicate plates. Mean, and SEM are plotted.Indicated cell lines grown for 72 h in complete medium or medium deficient in arginine and viability measured. Three independent experiments are shown with mean and SEM. Live cells (Annexin V−, 7‐AAD−) were analysed.Representative plot showing propidium iodide (RNASE‐PI) staining after culturing K562 cells with or without arginine for 72 h. Three independent experiments are shown with mean, and SD.Indicated cell lines grown for 16 h in complete medium or medium deficient in arginine and ASS1 protein levels were quantified. One representative plot is shown in top panel. Below, 3–4 independent experiments are shown with mean and SEM.ASS1 expression from the MILE study. The dotted line revers for average of Healthy Bone Marrow; ** refers to *P* < 0.0021, *** refers to *P* < 0.0002 and ****refers to *P* < 0.0001.ASS1 expression in indicated stem‐cell enriched datasets. CP: chronic phase, AP: accelerated phase, BP: blast phase. HPC and LPC refer to normal and leukaemic progenitor cells respectively. CMP, GMP, MEP and MPP refer to common myeloid, granulocyte‐macrophage, megakaryocyte‐erythrocyte, and multipotent progenitors respectively. Markers used in these studies were E‐MTAB‐2581: CD34, CD38, GSE47927E‐GEOD‐47927: CD34^+^, GSE43754E‐GEOD‐43754: CD34, CD38, ALDH‐high, E‐MEXP‐480: CD34, GSE24739: CD34, Hoechst, GSE15811E‐GEOD‐15811: CD34 transduced with indicated transgene. Data information: An unpaired *t*‐test was performed for statistical analysis for (A, B and D), a two‐way ANOVA with Ší'ák's multiple comparisons test was performed for (C), Kruskal‐Wallis test was performed on (E, F).

To determine the effect of amino acid depletion in a physiologically relevant setting we conducted a systematic dropout screen in Plasmax, a medium formulated based on the composition of human blood (Vande Voorde *et al*, 2019). This revealed that the non‐essential amino acids arginine, glutamine, serine, and tyrosine are required for proliferation of K562 cells (Fig 1A). Notably, even in the presence of citrulline and ornithine (both arginine precursors in the urea cycle), arginine‐deprivation had strong anti‐proliferative effects in CML cell lines (Fig 1A and B), with minimal effect on apoptosis (Fig 1C). Similar results were obtained in AML cells (Fig EV1A and B). In K562 cells, arginine deprivation caused the expected cell cycle disruption with an accumulation of cells in G2/M phase (Fig EV1C) (Alexandrou *et al*, 2018).

As the urea cycle enzyme ASS1 is upregulated in AML cells after arginine starvation (Crump *et al*, 2021), we assessed the effect of arginine deprivation in CML cell lines, using AML cell lines as a reference. CML and AML cell lines had variable levels of ASS1, which was upregulated following arginine deprivation (Fig EV1D). In contrast, neither primary CML nor normal CD34^+^ cells, had detectable ASS1 expression, even when compared with K562 cells which express low levels of ASS1 (Fig 1D). It is unlikely that BCR‐ABL1 is responsible for ASS1 suppression, as ASS1 was also not detectable by Western blotting in imatinib‐treated CD34^+^ CML cells (Fig 1D, all patient information is in Table EV1).

### Primary CML cells have low ASS1 gene expression compared with other leukaemia types

Given the undetectable ASS1 protein levels in primary normal and CML cells, we next examined ASS1 gene expression from the Microarray Innovations in Leukaemia (MILE) study (Haferlach *et al*, 2010; Bagger *et al*, 2016) (Figs 1E and EV1E). Here we found that CML and normal bone marrow (BM) cells have low ASS1 gene expression compared to other leukaemia types. As ASS1 expression levels are a known determinant of sensitivity to arginine deprivation, we subsequently examined ASS1 expression in indicated progenitor and stem‐cell enriched datasets that include chronic phase, accelerated phase and blast phase samples (Fig EV1F). While these studies use different markers such as CD34, CD38 (Zheng *et al*, 2006; Cramer‐Morales *et al*, 2013; Scott *et al*, 2016), Hoechst (Gerber *et al*, 2013), or CD34^+^ cells transduced with indicated BCR‐ABL1 transgenes (Agerstam *et al*, 2010), no significant differences in ASS1 levels were detected. This data would predict a consistent response to arginine deprivation across CML subtypes.

### Human CML CD34 ^+^ cells are arginine auxotrophic *in vitro* due to the absence of functioning urea cycle

**Figure EV2:**
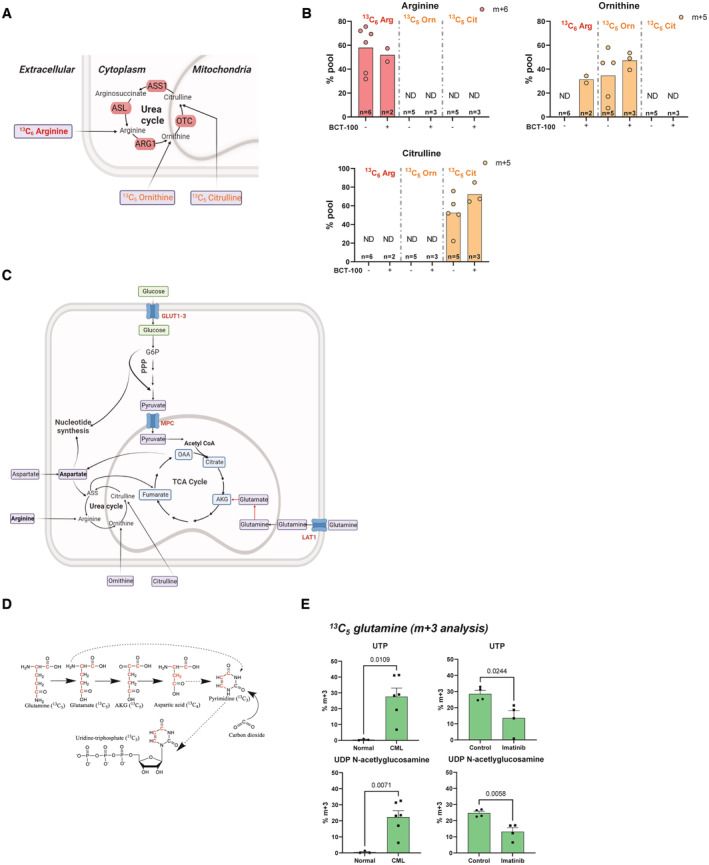
Primary CML samples lack functional urea cycle Schematic showing stable isotope tracing strategy for urea cycle enzymes that are abbreviated as follows; ARG: arginase, OTC: ornithine transcarbamylase, ASS1: arginosuccinate synthase 1, ASL: argininosuccinic acid lyase.Results from 48 h tracing of indicated labelled amino acid in CML CD34^+^ patient samples (^13^C_6_ arginine, ^13^C_5_ ornithine and ^13^C_6_ citrulline). Isotopologues are indicated in figure legend and *n*‐number on bar plots. Data is presented as mean and SEM.Diagram showing how generation of arginosuccinate can divert aspartate away from nucleotide synthesis.Schematic showing how ^13^C_5_ glutamine contributes to *de novo* synthesis of pyrimidines by donating three carbons to pyrimidines via aspartate.Analysis of ^13^C_5_ glutamine labelling in *de novo* synthesis of pyrimidines (*n* = 3 normal patient and *n* = 6 CML CD34^+^ samples for the left panel, *n* = 4 CML CD34^+^ patient samples for the right panel). An unpaired *t*‐test was performed for statistical analysis.

In line with the low ASS1 expression in primary CML cells, using stable‐isotope amino acid tracers (^13^C_6_, arginine, ^13^C_5_ ornithine, and ^13^C_5_ citrulline), we found that intracellular pools of urea cycle intermediates are entirely dictated by their presence in the environment, rather than by production through urea cycle enzymes (indicated by the lack of labelling in other cycle metabolites; Fig EV2A and B). As expected, treatment with recombinant human arginase BCT‐100 (catalyses conversion of arginine to ornithine) increased labelling in ornithine from labelled arginine (middle graph, second bar) (Cheng *et al*, 2007). However, there were no additional changes when arginine was depleted using BCT‐100, further suggesting absence of urea cycle activity.

**Figure 2:**
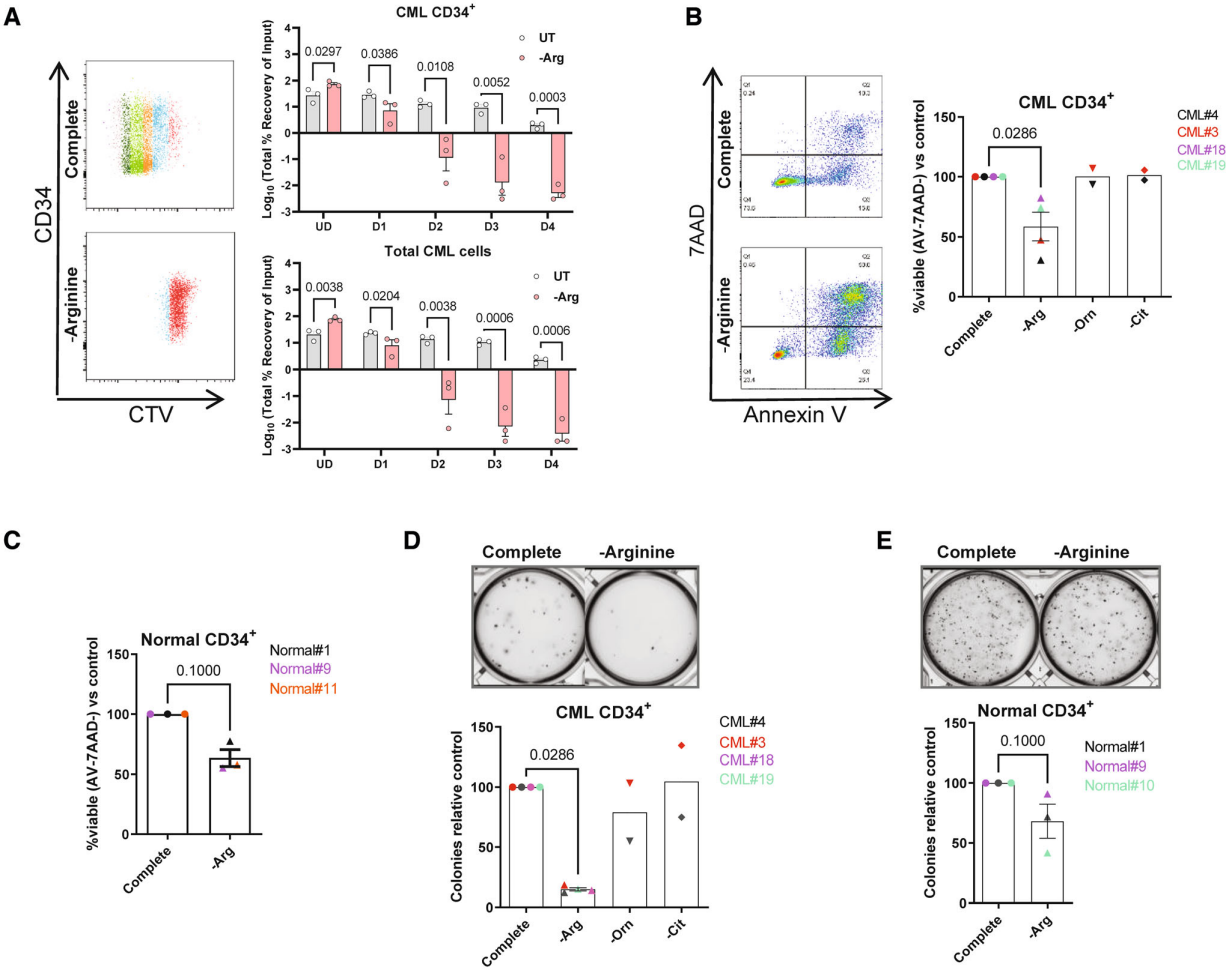
Primary CML CD34^+^ cells are highly sensitive to arginine deprivation CTV analysis of CML CD34^+^ patient samples after 72 h arginine starvation (*n* = 3). Data are shown with mean and SEM.Viability data from CML CD34^+^ patient samples recorded after 72 h starvation of indicated amino acid. Data are shown are from three biological replicates with mean and SEM plotted. Mean and SEM are shown.Viability from Normal CD34^+^ samples recorded after 72 h of arginine deprivation. Data shown are from three biological replicates. Mean and SEM are shown.CFCs from CML CD34^+^ patient samples generated after 72 h starvation of indicated amino acid. The upper panel shows representative image of colonies, counts are plotted in the lower panel. Data are shown are from four (‐arginine) or two (‐ornithine or ‐citrulline) biological replicates. Mean and SEM are shown.CFCs from Normal CD34^+^ samples generated after 72 h starvation of indicated amino acid. Upper panel shows representative image of colonies, counts are plotted in lower panel. Data are shown are from three biological replicates. Mean and SEM are shown. Data information: For statistical analysis, multiple unpaired tests with Benjamini, Krieger, and Yekutieli Benjamini two‐stage step up correction was performed out on log‐transformed data in (A), for (B–E) Mann–Whitney tests were performed. Source data are available online for this figure.

We next tested the effect of arginine‐deprivation on patient‐derived CML CD34^+^ cells. As with the CML cell lines, arginine‐deprivation caused a pronounced block in proliferation in primary cells (Fig 2A). However, in contrast to the cell lines, arginine deprivation caused a significant induction of apoptosis and a substantial decrease in clonogenicity in CML CD34^+^ cells, which was not observed following ornithine or citrulline withdrawal (Fig 2B and D). Importantly, we saw less effect on the viability and clonogenicity in normal CD34^+^ cells (Fig 2C and E).

It has been reported that maintaining low ASS1 levels can facilitate rapid proliferation as ASS1 diverts aspartate away from *de novo* nucleotide synthesis (Rabinovich *et al*, 2015; Garcia‐Bermudez *et al*, 2018; Qi *et al*, 2021) (Fig EV2C). Therefore, we measured *de novo* synthesis of pyrimidines using ^13^C_5_ glutamine (Fig EV2D). We discovered significant upregulation of *de novo* pyrimidine synthesis in CML CD34^+^ cells compared to normal CD34^+^ cells, perhaps due to higher proliferation rate and requirement for nucleotide synthesis, and imatinib treatment caused only a partial reduction (Fig EV2E). Pyrimidines are also used for protein glycosylation, and we observed similar results for a glycosylation pathway intermediate (Fig EV2E, UDP‐N‐acetylglucosamine).

It is important to note that changes to metabolic demands or proliferation that can alter the requirement of arginine to normal cells, such as T‐cells, is context dependent such as reported during immune therapy (Mussai *et al*, 2019). As such, it is possible that normal cells would become similarly sensitive during haemopoietic expansion following myeloablative treatment. Additionally, Crump *et al* (2021) showed that elevated levels (150 μM) of plasma citrulline found in AML patients can support the growth of AML cell lines via ASS1 upregulation following arginine depravation. However, citrulline levels in CML patients remain to be determined.

### Pharmacological arginine depletion selectively targets human CML CD34 ^+^ cells

**Figure 3:**
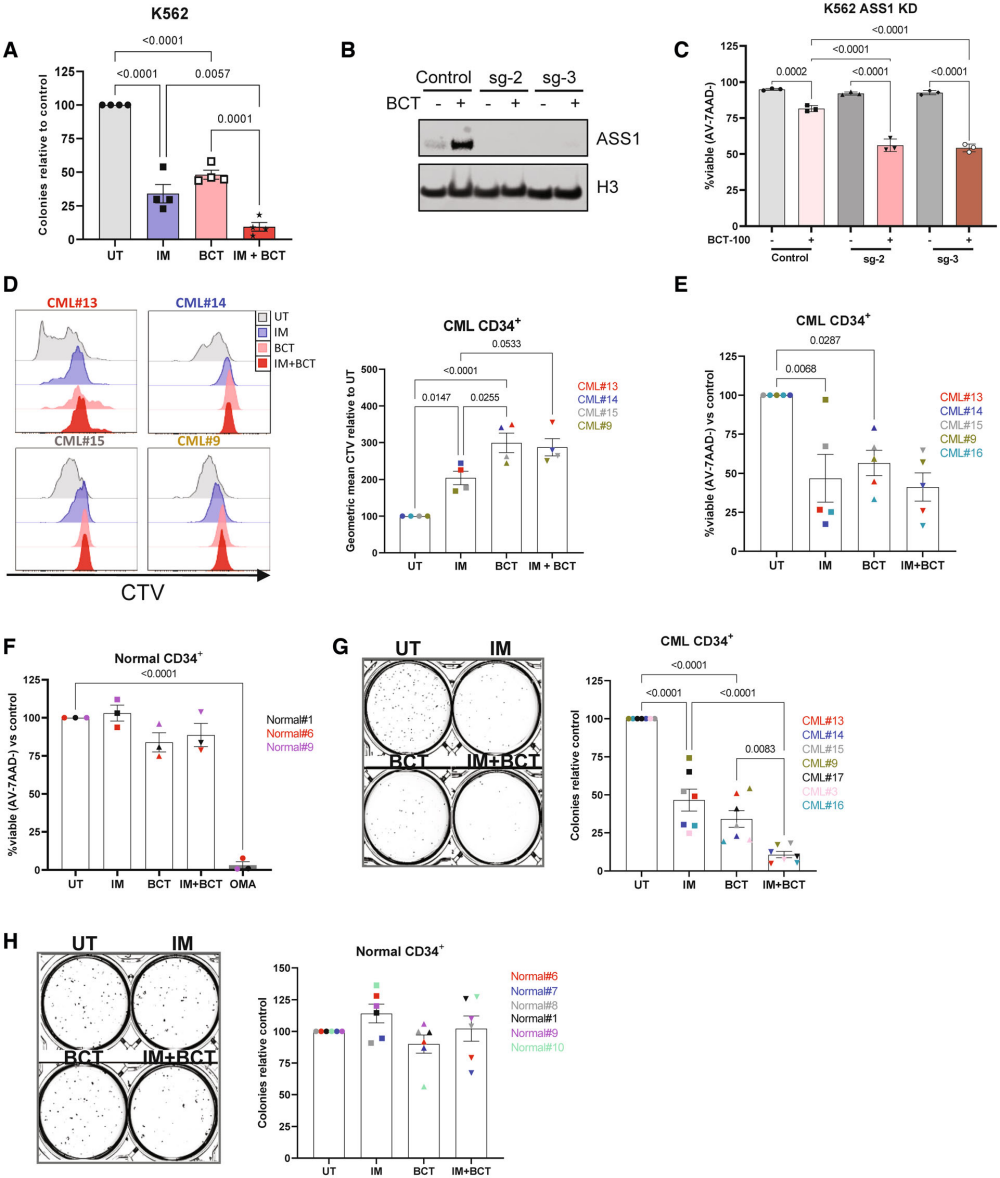
CML CD34^+^ cells are sensitive to pharmacological arginine depletion K562 cells were grown for 72 h in imatinib (600 nM), BCT‐100 (1,000 ng/ml) or combination before seeding for CFCs. CFCs from four independent experiments are shown. Mean and SEM are shown.Indicated cell lines (Control or knock‐down (sg‐2 or sg‐3)) grown for 24 h in absence or presence of BCT‐100 (1,000 ng/ml) and ASS1 protein levels measured.Indicated cell lines (Control or knock‐down (sg‐2 or sg‐3)) grown for 72 h in absence or presence of BCT‐100 (1,000 ng/ml) and viability measured. Three independent experiments are shown with mean and SD. Live cells (Annexin V−, 7‐AAD−) were analysed.CellTrace violet (CTV) proliferation profiles from four CML CD34^+^ patient samples are shown (four biological replicates). Cells were grown for 72 h in imatinib (2 μM), or BCT‐100 (100 ng/ml) before flow cytometry analysis. Geometric means are plotted with Mean and SEM.Viability data from CML CD34^+^ patient samples (five biological replicates) recorded after 72 h of treatment as in (D), Mean and SEM are shown.Viability from Normal CD34^+^ samples (three biological replicates) recorded after 72 h of treatment as in (D). Mean and SEM are shown.CFCs from CML CD34^+^ samples are shown. Cells were treated as in (D) before seeding for CFCs. The left panel shows representative image of colonies, counts are plotted in the right panel. Data are shown are from seven biological replicates. Mean and SEM are shown.CFCs from three normal CD34^+^ samples are shown. Cells were treated as in (D) before seeding for CFCs. The left panel shows representative image of colonies, counts are plotted in the right panel. Data are shown are from six biological replicates. Mean and SEM are shown. Data information: For statistical analysis, ordinary one‐way ANOVA with Tukey's correction for multiple comparisons was performed in (A–H). Source data are available online for this figure.

**Figure EV3:**
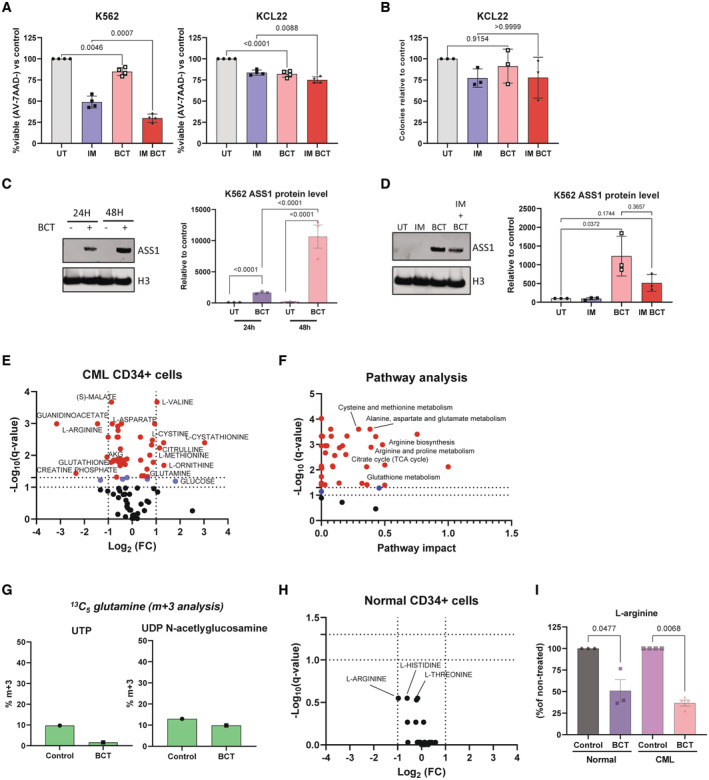
ASS1 is required for CML cell lines to escape apoptosis induced by arginine starvation K562 or KCL22 cells were grown for 72 h in imatinib (600 nM), BCT‐100 (1,000 ng/ml) or combination and viability measured. Data from four independent experiments are shown. Mean and SEM are shown.KCL22 cells were treated as in (A) before seeding for CFCs. Data from three independent experiments are shown. Mean and SEM are shown.Western blotting was used to visualise ASS1 protein levels in K562 cells exposed to BCT‐100 (1,000 ng/ml) for indicated times. On the left a representative blot is shown. The right panel shows data from three independent experiments. Mean and SEM is plotted.Western blotting was used to visualise ASS1 protein levels in K562 cells as treated in Fig 3A. The left panel shows a representative blot. The right panel shows data from three independent experiments. Mean and SEM is plotted.Volcano plot from LC–MS analysis of CML CD34^+^ cells (*n* = 4 patients) treated with BCT‐100 (100 ng/ml) for 24 h. Blue denotes *q*‐value < 0.1, red denotes *q*‐value < 0.05.Pathway analysis of (E). Blue denotes *q*‐value < 0.1, red denotes *q*‐value < 0.05 (Benjamini & Hochberg).Analysis of ^13^C_5_ glutamine labelling in *de novo* synthesis of pyrimidines one CML CD34^+^ samples that was treated with BCT‐100 (100 ng/ml) for 24 h.Volcano plot from LC–MS analysis of normal CD34^+^ cells (*n* = 3 biological samples) treated with BCT‐100 (100 ng/ml) for 24 h.L‐arginine from (E) (4 patient samples) and (H) (*n* = 3 biological samples). Mean and SEM is plotted. Data information: An ordinary one‐way ANOVA with Tukey's correction for multiple comparisons were performed on data from (A–C) (data from (C) was log‐transformed to ensure normality). Kruskal‐Wallis test with the Benjamini and Hochberg false discover correction was used to analyse data for (D). Metabolanalyst was used to calculate *q*‐values after mean‐centering and R‐Log transforming data for (E) and (H). For (F), pathway analysis was conducted using Metabolanalyst after mean‐centering and R‐Log transforming data. The Globaltest and relative betweenness centrality were used on Homo sapiens KEGG database. A Kruskal‐Wallis test was used to analyse data for (I).

While dietary restriction can lower arginine levels, this is inferior to recombinant enzymes such as BCT‐100, which reduces arginine to non‐detectable levels in blood (Yau *et al*, 2013). BCT‐100 significantly reduced the viability of ASS1‐low K562 cells, with a further reduction observed in the presence of imatinib (Fig EV3A). The effect was less pronounced in ASS1‐high KCL22 cells (Fig EV3A). BCT‐100 significantly reduced the clonogenicity of ASS1‐low K562 cells, with a further reduction observed in the presence of imatinib (Fig 3A). These reductions were absent in ASS1‐high KCL22 cells (Fig EV3B). We subsequently examined ASS1 levels in BCT‐100‐treated K562 cells. BCT‐100 caused a time‐dependent increase in ASS1 levels (Fig EV3C), while this was partially reduced in the presence of imatinib, although this did not reach statistical significance (Fig EV3D). To further examine the role of ASS1 in CML we generated ASS1 knock‐down (KD) K562 cells (Fig 3B). While there was no difference in viability between control and KD cells, ASS1‐deficient cells had decreased viability in the presence of BCT‐100 (Fig 3C).

We next treated patient‐derived CML CD34^+^ cells with imatinib, BCT‐100 and the combination. Similar to arginine starvation, BCT‐100 treatment potently blocked proliferation of CML CD34^+^ cells to a greater extent than imatinib single treatment (Fig 3D). As a single agent, BCT‐100 significantly reduced viability of CML CD34^+^ cells, with a similar effect being observed in combination with imatinib (Fig 3E). No significant effect of BCT‐100 treatment was observed on normal CD34^+^ cells, in contrast to cells treated with omacetaxine mepesuccinate (OMA), an inhibitor of protein biosynthesis, used on occasion for advanced phases of CML (Fig 3F). As with K562 cells, BCT‐100 caused a significant reduction in the clonogenicity of CML CD34^+^ cells with a further reduction in combination with imatinib (Fig 3G). In contrast, BCT‐100 treatment had no significant effect on the clonogenicity of normal CD34^+^ cells (Fig 3H). It is important to note that the effective dose for CML CD34^+^ cells (100 ng/ml) is less than what was required to see an effect in cell lines (1,000 ng/ml).

As single amino acid restriction can have wider effects on intracellular metabolism, we tested the metabolic effects of BCT‐100 on primary CML CD34^+^ cells. BCT‐100 treatment caused perturbation of multiple metabolites (Fig EV3E). Pathway analysis revealed deregulation of amino acid catabolic pathways, redox metabolism, and the tricarboxylic acid cycle (Fig EV3F), as previously reported (Changou Chun *et al*, 2014). Notably, 16/18 metabolites with increased intracellular abundance upon BCT‐100 treatment were medium components, indicating decreased metabolic processing. In line with this, most metabolites with decreased abundance were metabolic intermediates (Table EV2). Subsequent analysis of ^13^C_5_ glutamine into pyrimidines in BCT‐100 treated CML samples showed that this was decreased (Fig EV3G). However, as steady‐state levels of most metabolic intermediates were decreased, we cannot preclude that the incorporation of other carbon sources that produce aspartate are also reduced. Finally, we performed additional experiments on normal CD34^+^ cells. While the changes here failed to reach significance following multivariate analysis (Fig EV3H), when examining L‐arginine levels, we confirmed a consistent decrease in both normal and CML datasets (Fig EV3I).

### BCT‐100 treatment causes transcriptional changes in low ASS1 expressing primary CML cells

**Figure 4:**
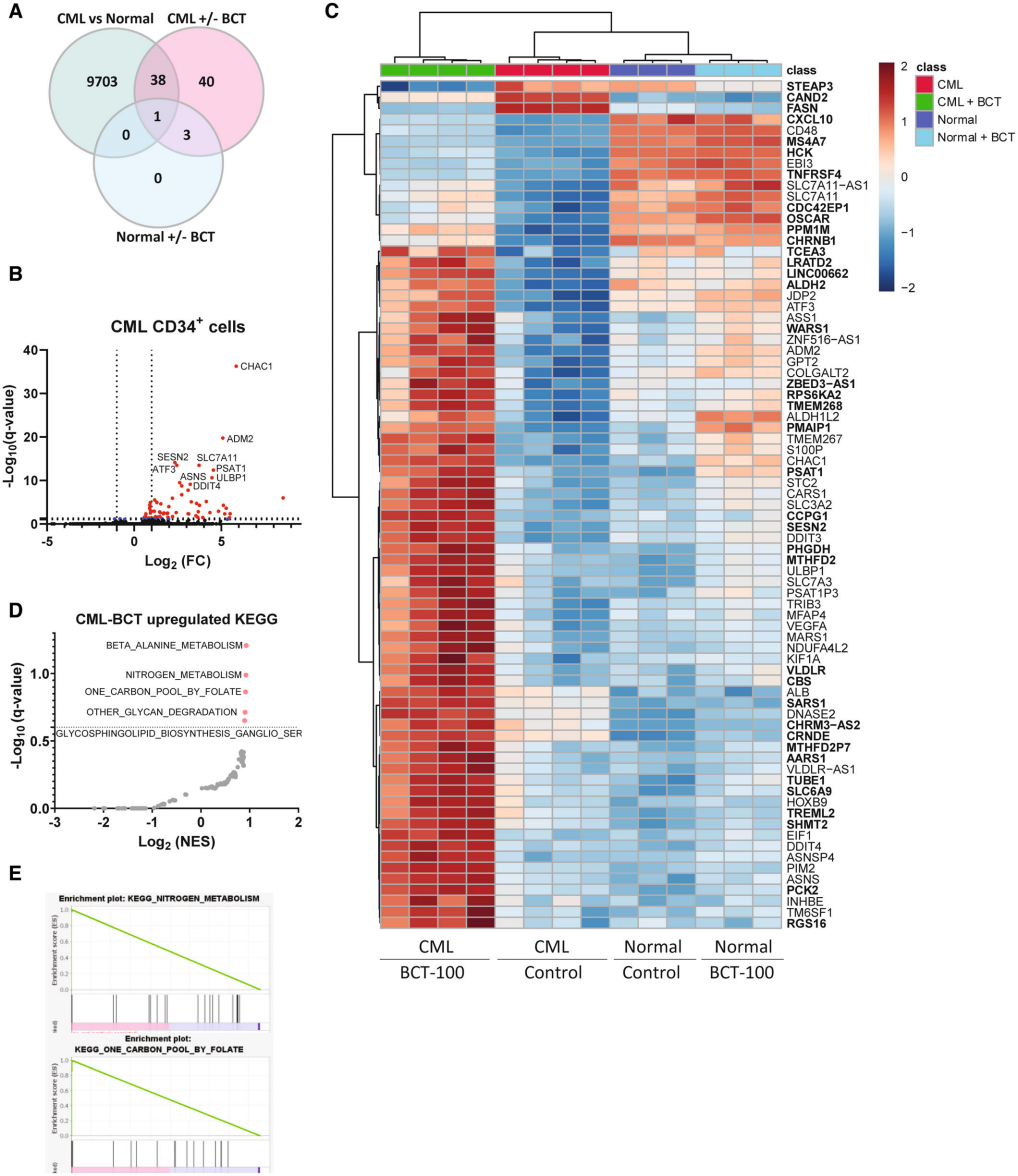
ASS1 knockout phenocopies CML's sensitivity to arginine deprivation Transcriptomics analysis was conducted on normal or CD34^+^ CML cells that were treated for 24 h with BCT‐100 (100 ng/ml). Venn diagram showing differentially expressed genes from indicated comparisons.Volcano plot showing differentially expressed (DE) genes between CML CD34^+^ vehicle and BCT‐100 treated cells, red denotes *q*‐value < 0.1.Heatmap showing DE genes from (B). In bold are genes that were also DE comparing normal to CML.Upregulated KEGG pathways from GSEA analysis are shown. In red are sets meeting false discovery rate < 0.25.Indicated plots from (D) are shown. Ordinary one‐way ANOVA with Tukey's correction for multiple comparisons were performed on (A). Data information: For (A–D), DESEQ2 and GSEA were used as described in methods. Source data are available online for this figure.

**Figure EV4:**
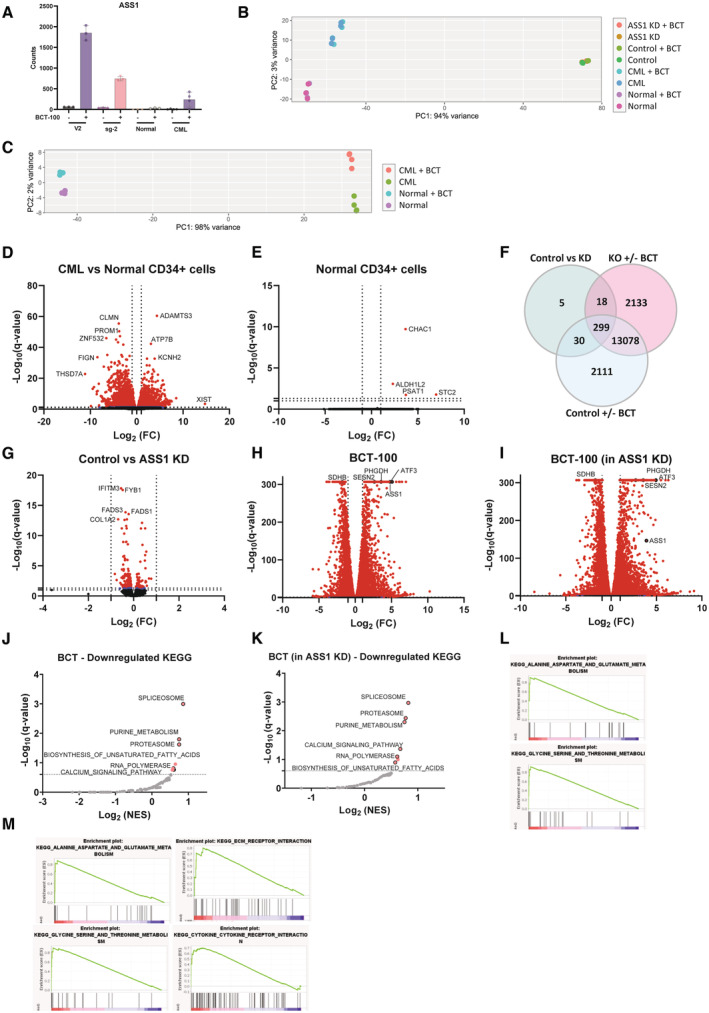
Pharmacological arginine depletion uniqely effects CML patient samples with drastic effects on both control and ASS1 KO cells Indicated cell lines, normal or CML CD34^+^ cells were treated for 24 h with BCT‐100 (1,000 ng/ml for cell lines and 100 ng/ml for primary samples). ASS1 counts from indicated cell lines (three independent experiments), normal samples (*n* = 3 biological replicates) and CML patient samples (*n* = 4 biological replicates) are shown. Mean and SD is plotted.PCA for samples in (A).PCA for primary samples only.Volcano plot showing differentially expressed genes between normal and CML CD34^+^ cells, red denotes *q*‐value < 0.05, blue denotes *q*‐value < 0.1.Volcano plot showing differentially expressed genes between normal CD34^+^ vehicle and BCT‐100 treated cells, red denotes *q*‐value < 0.05.Venn diagram showing differentially expressed genes from indicated comparisons.Volcano plot showing differentially expressed genes between Control and ASS1 KD K562 cells, red denotes *q*‐value < 0.05, blue denotes *q*‐value < 0.1.Volcano plot showing differentially expressed genes between Control K562 cells treated with BCT‐100 or vehicle, red denotes *q*‐value < 0.05, blue denotes *q*‐value < 0.1.Volcano plot showing differentially expressed genes between ASS1 KD K562 cells treated with BCT‐100 or vehicle, red denotes *q*‐value < 0.05, blue denotes *q*‐value < 0.1.Downregulated KEGG pathways from GSEA analysis on genes from the instersect of KD‐BCT and Control‐BCT are shown. Here pi values (computed by multiplying log_2_ fold change by −log_10_ (*q*‐value)) from Control‐BCT were used. In red are sets with corrected *P*‐values less than false discovery rate threshold (*P* < 0.25: dotted line).Downregulated KEGG pathways from GSEA analysis on genes from the instersect of KD‐BCT and Control‐BCT are shown. Here pi values from KD‐BCT were used. In red are sets with corrected *P*‐value less than false discovery rate threshold (*P* < 0.25: dotted line).The upregulated pathways corresponding to (J) are shown.The upregulated pathways corresponding to (K) are shown. DESEQ2 and GSEA were used as described in methods.

To further investigate the cellular response to arginine depletion, we performed RNA sequencing (RNA‐seq) on the engineered K562 cells as well as primary normal and CML CD34^+^ cells, in the absence or presence of BCT‐100. Despite visible reduction at the protein levels, we observed an increase in ASS1 mRNA levels in BCT‐100‐treated KD K562 cells, albeit less than in control cells. Notably, the increase in ASS1 following BCT‐100 treatment in CML CD34^+^ cells was less compared to K562 KD cells (Fig EV4A). Principal component analysis (PCA) revealed that the primary cells clustered far apart from the cell lines (Fig EV4B), thus we analysed primary samples separately. Here, PCA showed that the largest differences were between CML and normal cells, with treatment causing less changes (Fig EV4C). Differential expression analysis confirmed that most significantly differentially expressed genes were between CML and normal, with BCT‐100 altering levels of 82 genes in CML cells and only four in normal cells (Figs 4A and B, and EV4D and E). The majority of differentially expressed genes in BCT‐treated CML were upregulated, including ASS1 and arginine transporter SLC7A3 (CAT3) (Fig 4C). Other upregulated genes included those related to translation (ATF3, EIF1) and serine metabolism (PSAT1, ALDH1L2, PHGDH, MTHFD2 and SHMT2). In agreement with this, gene set enrichment analysis (GSEA) showed that upregulated pathways included one carbon metabolism and nitrogen metabolism (Fig 4D and E). These findings agree with previous studies demonstrating that arginine deprivation leads to upregulation of serine biosynthesis in ASS1‐deficient breast, melanoma, and sarcoma cell lines (Kremer *et al*, 2017; Cheng *et al*, 2018). In contrast to the primary samples, BCT‐100 caused the majority of changes in the K562 cell line, irrespective of ASS1 levels (Fig EV4F–I) with both control and KD having similar pathways deregulated (Fig EV4J–M). Further studies will be needed to define the role of upregulated serine biosynthesis in arginine‐deprived CML cells and if this upregulation is evident in patients with leukaemia or other ASS1‐deficient cancers.

### Pharmacological arginine depletion eradicates human CML CD34 ^+^ CD38 ^−^ LSCs *in vivo*

**Figure 5:**
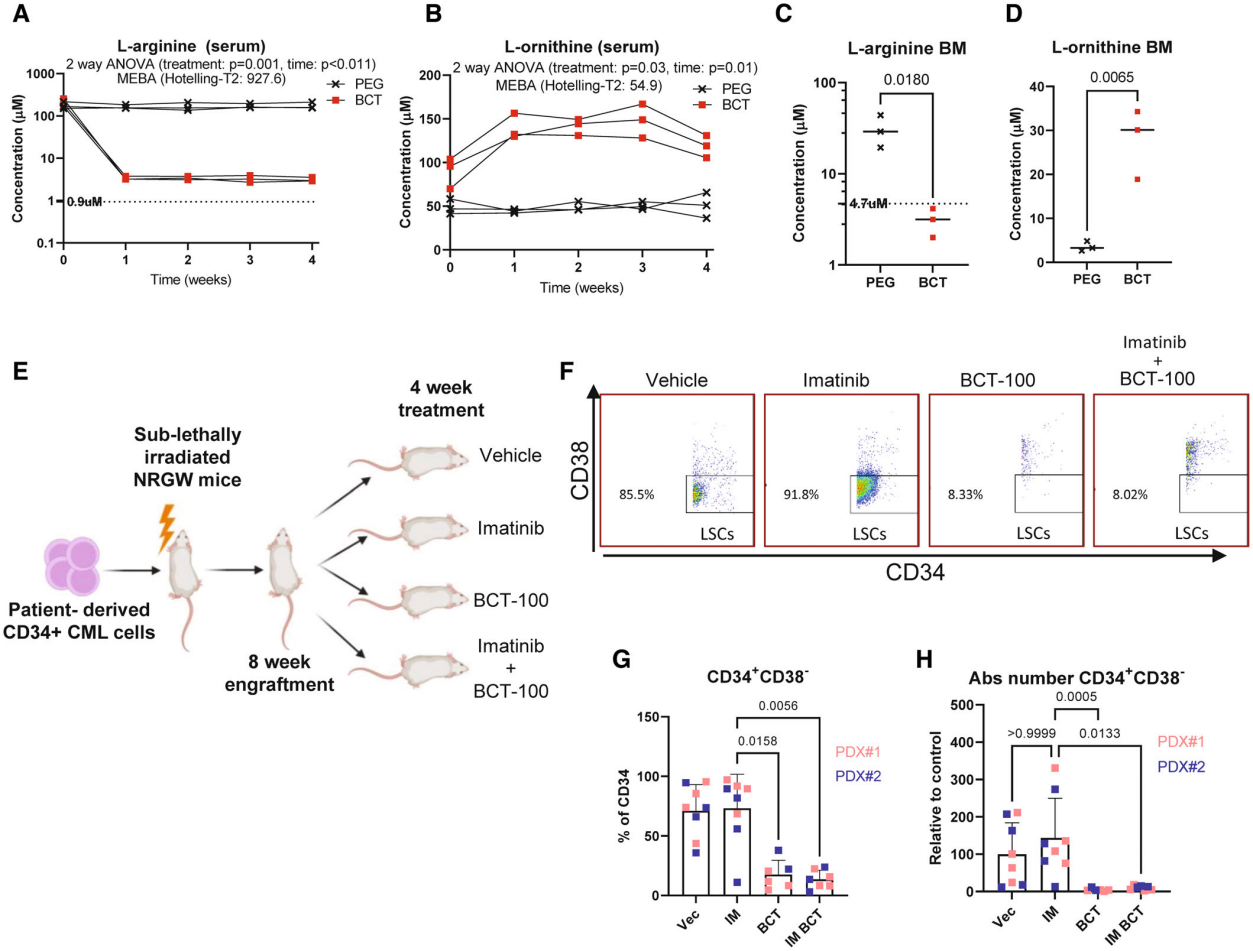
CML CD34^+^CD38^−^ LSCs cells are sensitive to pharmacological arginine depletion *in vivo* A–DAbsolute concentration of indicated amino acid in serum or bone marrow (BM) is shown. Dashed line denotes lowest linear point from standard curve.EExperimental outline of PDX experiment with four arms; Vehicle: *n* = 8 mice, Imatinib: *n* = 8 mice, BCT‐100: *n* = 6 mice, Combo: *n* = 7 mice.FRepresentative data showing CD34^+^CD38^−^ population from each treatment group.GThe percentage of CD34^+^CD38^−^ cells (from CD34^+^) is shown. Biological replicate data from all mice, average and SD are plotted. Vehicle: *n* = 8 mice, Imatinib: *n* = 8 mice, BCT‐100: *n* = 6 mice, Combo: *n* = 7 mice.HThe absolute number of CD34^+^CD38^−^ cells is shown. Biological replicate data from all mice, average and SD are plotted. Vehicle: *n* = 8 mice, Imatinib: *n* = 8 mice, BCT‐100: *n* = 6 mice, Combo: *n* = 7 mice. Data information: Metabonalyst was used to conduct both two‐way ANOVA and Multivariate Empirical Bayes Analysis (MEBA) on R‐Log transformed data in (A) and (B). an unpaired *t*‐test was performed for (C) and (D). A Kruskal‐Wallis test was used to analyse data in (G) and (H). Source data are available online for this figure.

**Figure EV5:**
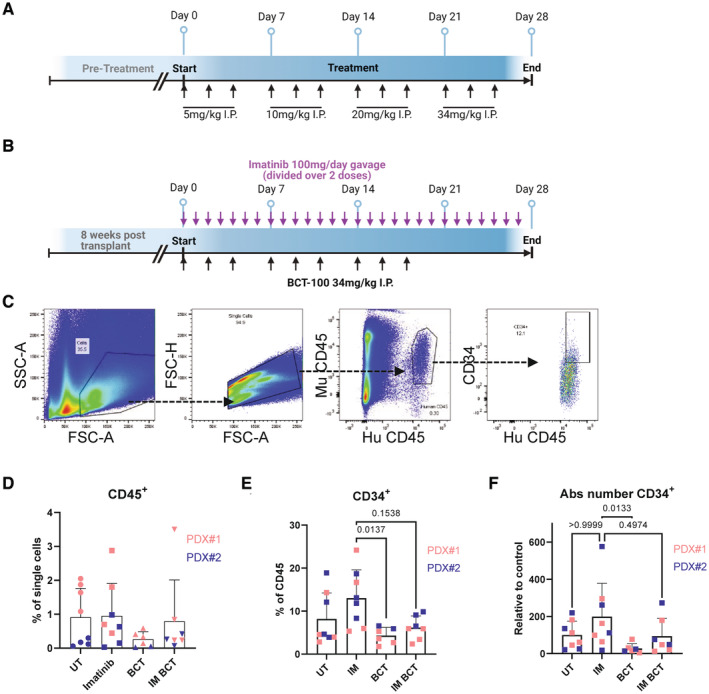
Pharmacological arginine depletion is human effective in CML LSC xenograft model Dosing strategy for escalation study. Serum samples were taken prior to first dose, each first escalation dose and 24 h after last dose.Dosing strategy for PDX experiment.Gating strategy is shown for flow cytometry analysis.The percentage of CD45^+^ cells from single cells is shown. Biological replicate data from all mice, average and SD are plotted. Vehicle: *n* = 8 mice, Imatinib: *n* = 8 mice, BCT‐100: *n* = 6 mice, Combo: *n* = 7 mice.The percentage of CD34^+^ cells (from CD45) is shown. Average and SD are plotted. Biological replicate data from all mice, average and SD are plotted. Vehicle: *n* = 8 mice, Imatinib: *n* = 8 mice, BCT‐100: *n* = 6 mice, Combo: *n* = 7 mice.The absolute number of CD34^+^ cells is shown. Average and SD are plotted. Biological replicate data from all mice, average and SD are plotted. Vehicle: *n* = 8 mice, Imatinib: *n* = 8 mice, BCT‐100: *n* = 6 mice, Combo: *n* = 7 mice. Data information: A Kruskal‐Wallis test was used to analyse data for (D–F) with significant changes (adjusted *P*‐value, all *P*‐values in (D) are > 0.5) or relevant comparisons are shown.

We subsequently tested the effect of BCT‐100 *in vivo* following a dose escalation pilot experiment (Fig EV5A). Pharmacodynamic analysis using liquid chromatography‐mass spectrometry (LC–MS) revealed that BCT‐100, which was well tolerated, significantly lowered serum arginine levels, with a corresponding increase in ornithine at all doses (Fig 5A and B). As the effectiveness of BCT‐100 treatment in the BM niche is unknown, we conducted LC–MS on endpoint samples from which we rapidly isolated BM fluid (Amend *et al*, 2016). BCT‐100 decreased BM serum arginine levels (below LC–MS detection limit) with a corresponding increase in ornithine (Fig 5C and D).

Finally, we used the patient‐derived xenograft model to test the effectiveness of BCT‐100 on BM‐located human CML LSCs *in vivo*. While mice in this model do not develop lethal disease, the model is the gold standard for assessment of human CML LSC survival as the duration of engraftment ensures that cells present at end of treatment are LSCs or LSC‐derived (Abraham *et al*, 2016; Kuntz *et al*, 2017; Ianniciello *et al*, 2021). Here, chronic phase CML CD34^+^ cells were transplanted into irradiated NRGW mice (Miller *et al*, 2017). After 8 weeks, mice were randomised into treatment groups: vehicle, imatinib (50 mg/kg; BID), BCT‐100 (34 mg/kg; 3×/week) and combination (Figs 5E and EV5B). After 4 weeks treatment, BM was analysed by flow cytometry (Fig EV5C). While the percentage of human CD45^+^ cells was variable (Fig EV5D), there was a decreasing trend in percentage and absolute number of CD45^+^CD34^+^ cells in BCT‐100‐treated mice, with no significant difference between BCT‐100 and combo‐treated mice (Fig EV5E and F). However, BCT‐100 caused a drastic reduction in the more primitive CD45^+^CD34^+^CD38^−^ LSC population (Fig 5F and G), which was even more pronounced when the absolute number of LSCs were calculated (Fig 5H).

In conclusion, we report for the first time that primitive human CML cells are sensitive to arginine depletion both *in vitro* and *in vivo*. Notably this effect was most evident in the BM‐located LSC population that is intrinsically TKI‐resistant (Hamilton *et al*, 2012). Critically, ASS1 transcript levels are consistently low in primary CML cells (including LCSs) irrespective of disease phase, which suggests that it is an arginine auxotrophic leukaemia type. This is the first instance that clinically relevant pharmacological arginine depletion has been demonstrated to target therapy‐resistant LSCs, highlighting BCT‐100 treatment as a viable strategy to improve current standard of care for CML.

## Materials and Methods

### Statistics

No statistical methods were used to determine sample size. The investigators were not blinded to samples or treatments during experiments. For patient‐derived xenograft (PDX) experiments, mice were randomly assigned to treatment groups. Statistical tests were calculated using Graphpad Prism (v9) or MetaboAnalyst 5.013 as denoted in figure legends. Indicated *P*‐values are plotted, if not shown for a given comparison, result was non‐significant (*P* > 0.05).

### Cell culture

Primary CML samples were thawed and recovered overnight using physiological medium (Plasmax) (Vande Voorde *et al*, 2019). This medium was supplemented with labelled or non‐labelled nutrients as well as standard supplements and growth factors as described previously (Kuntz *et al*, 2017), then filter sterilised through a 0.2 μM filter (Fisher Scientific: 10509821). Stable isotope tracers were purchased from Cambridge Isotopes (^13^C_6_ Arginine: Cat# CLM‐2265, ^13^C_5_ Ornithine: Cat# CLM‐4724 and ^13^C_5_ Citrulline: Cat# CLM‐8653) and added at concentrations found in Plasmax. Primary samples were seeded at a density of 400,000 cells/ml and cell lines at 100,000 cells/ml. For cell lines, the medium was supplemented with 10% dialysed FBS (Thermo Fisher Scientific Cat# A33820‐01). Imatinib was purchased from LC Laboratories and BCT‐100 was supplied from BCT International. The CD34^+^ cells were isolated using the CliniMACS (Miltenyi Biotec) to 95% purity while normal CD34^+^ cells (> 90% purity) using human CD34 MicroBeads (Miltenyi Biotec: Cat# 130‐100‐453), according to manufacturer's instructions.

### RNA extraction, library prep, sequencing, and analysis

RNA for cell lines was extracted with an RNAeasy Kit (Qiagen Cat# 74104), while RNA from primary cells was extracted with the Arcturus™ PicoPure™ RNA Isolation Kit (Thermo Fisher Scientific Cat# KIT0204), with DNA removed using the DNase Set‐RNase free (Qiagen Cat# 79254) following manufacturer instructions. Libraries were prepared with the Stranded mRNA Prep kit (Illumina). Samples were sequenced on Illumina NextSeq 500 using the High Output 75 cycles kit (2 × 36 cycles, paired end reads, dual index; Illumina) to obtain minimum 20 million reads per sample. Subsequently, FastQ files were generated using Illumina's bcl2fastq (v. 2.20.0.422). The sequencing data has been deposited on GEO with accession GSE226887. QC was conducted using fastqc v0.11.8, reads trimmed using trimgalore v0.4.4. Trimmed reads were then aligned to GRCh38 using Hisat2 v2.1.0 and sorted into bam files using samtools (v1.15.1.5). The bam files for two independent runs of each samples were merged using samtools (v1.15.1.5). Read count extraction was performed using featureCounts function in Subread package (v2.0.1) and resulting featureCounts analysed using DESEQ2 (v1.34.0). GSEA (version 4.1) was used on pre‐ranked lists (ranked by pi score that was computed by multiplying log_2_ fold change by −log_10_ (corrected *P*‐value)).

### Generation of ASS1 knockdown (CRISPR‐CAS9) cell line

To target the human ASS1 gene, guides were designed using the genscript tool https://www.genscript.com/gRNA‐database.html. Two guides were ordered from Integrated DNA Technologies. These were then annealed and cloned in Bsmb I–digested lentiCRISPRv.2‐puro (RRID: Addgene_52961). After stable integration of lentiCRISPRv.2 using lentiviral transfection and 1‐week selection using puromycin (2.5 μg/ml), knockdown was validated by performing western blotting. Oligonucleotides sequences are shown below with relevant targeting sequences in bold and underlined:

sg2 forward: CACCG **CCATGCTCATTTAGACATCG**

sg2 reverse: AAAC **CGATGTCTAAATGAGCATGGC**

sg3 forward: CACCG **CCTCGATGTCTAAATGAGCA**

sg3 reverse: AAAC **TGCTCATTTAGACATCGAGGC**

### Lentivirus production

Lentiviruses for pLentiCRISPRv.2 were produced by the calcium phosphate method using pCMV‐VSV‐G (envelope plasmid: RRID: Addgene_8454) and psPAX2 (packaging constructs: RRID: Addgene_12260) vectors and human embryonic kidney (HEK) 293FT cells for transfection.

### Western blot analysis

Chronic phase CD34^+^ CML cells were lysed in RIPA buffer (Thermo Fisher Scientific Cat# 89900) containing mini‐Complete protease inhibitor cocktail and phosphatase inhibitors (Roche Cat# 04906837001 and Cat# 04693132001). Total protein concentration was quantified using a Pierce BCA kit (Thermo Fisher Scientific Cat# 23227). Equal amounts of protein (5 μg) were heated at 95°C for 5 min and separated (120 V) in 4–12% gels (Thermo Fisher Scientific Novex Cat# NP0321BOX) for SDS–PAGE. Proteins were transferred onto PVDF membranes (Thermo Fisher Scientific Cat# 21882), blocked in 2% BSA (in Tris‐buffered saline, 0.01% Tween (TBS‐T)) for 1 h. Membranes were then incubated overnight at 4°C with the primary antibodies (1:1,000), rinsed three times with TBS‐T, then incubated with secondary HRP‐linked antibodies (1:10,000) for 1 h at room temperature. The SuperSignal West Femto Maxi was used to detect proteins (Thermo Fisher Scientific: 34095) and imaging carried out using a LI‐COR Odyssey Fc gel‐doc system. Antibodies used were Histone 3 (Active Motif Cat# 39763), ASS1 (Cell Signalling Technology Cat# 70720), anti‐rabbit IgG HRP‐linked Ab (Cell Signalling Technology Cat# 7074) and anti‐mouse IgG HRP‐linked Ab (Cell Signalling Technology Cat# 7076).

### Metabolic studies

Primary sample preparation and LC–MS were conducted as previously described (Kuntz *et al*, 2017), while analysis was conducted using Tracefinder 4.1 (Thermo Fisher Scientific). Serum samples were prepared by allowing samples to clot at room temperature (20–30 min), centrifugation (2,000 *g*, 4°C, 20 min) and extracted 1:50 in ice‐cold solvent. BM extracellular samples were prepared as described previously (Amend *et al*, 2016), with the exception being 1 tibia, hip, and femur was spun into 50 μl and supernatant extracted 1:50. Quantification was carried out using internal (within pooled sample) standard curve of stable‐isotope labelled versions of metabolites.

### Colony‐forming assay

Primary cells were plated in above medium in the presence of the indicated drugs. After 72 h, cells from each condition were transferred to methylcellulose enriched with human cytokines (Bio‐Techne Cat# HSC005) in duplicate, and colonies were manually counted after 12–14 days. Cell lines were cultured in Plasmax supplemented with 10% dialysed FBS (Thermo Fisher Scientific Cat# A33820‐01) and 1% penicillin/streptomycin and base methylcellulose (Bio‐Techne Cat# HSC002) used for colony‐forming cell (CFC) assays.

### CTV, RNASE‐PI and apoptosis

CellTrace™ Violet Cell Proliferation Kit, for flow cytometry (Thermo Fisher Scientific Cat# C34557) was used according to manufacturer's instructions. Subsequently cells were stained with Annexin V (FITC or APC, BioLegend: 640906 or 640941, 5 μl/test), 7‐AAD (BD Bioscience: 559925, 5 μl/test) and CD34^+^ (APC, BD Bioscience: 555824, 2 μl/test) in 50 μl HBSS buffer for 20 min. For RNASE‐PI staining (Thermo Fisher Scientific: F10797), 200,000 cells were fixed in ethanol then stained with 100 μl for 20 min. Data was acquired using a BD FACSVerse flow cytometer, and data analysed using Flo Jo (V10).

### PDX experiments

One million chronic phase CD34^+^ CML cells were transplanted I.V. into sub‐lethally irradiated (2.5 Gy) female NOD.Cg‐Rag1^tm1Mom^ Kit^W‐41J^ Il2rg^tm1Wjl^/EavJ NSG mice (8–10 weeks old) (Jackson Laboratory). Eight weeks post‐transplant, drug treatment was started with both imatinib (50 mg per kg body weight; oral gavage twice daily for 4 weeks) and BCT‐100 (30 mg/kg; I.P. 3 times/week for 3 weeks). At the endpoint, BM cells were collected as described previously (Amend *et al*, 2016). Cells were stained with anti‐mouse CD45 (APC‐Cy7 BD Biosciences: Cat# 557659, RRID: AB_396774), anti‐human CD45 (FITC; BD Biosciences: Cat# 555482, RRID: AB_395874), anti‐human CD34 (APC; BD Biosciences: Cat# 555824, RRID: AB_398614), and anti‐human CD38 (PerCP; BioLegend: Cat# 303520, RRID: AB_893313) antibodies prior to flow cytometry data acquisition using a BD FACSVerse flow cytomer with data analysis using FlowJo Software.

### Study design

#### Ethics

Chronic myeloid leukaemia patient samples were obtained from peripheral blood or leukapheresis product. Patients were in chronic‐phase CML at the time of diagnosis, gave written informed consent in agreement with the Declaration of Helsinki and the approval of the National Health Service (NHS) Greater Glasgow and Clyde Institutional Review Board. Ethical approval has been granted to the research tissue bank (REC 15/WS/0077) and for use of surplus human tissue in research (REC 10/S0704/60). Normal CD34^+^ cells were isolated from femoral head material.

## Author contributions

**Kevin M Rattigan:** Conceptualization; data curation; formal analysis; investigation; visualization; writing – original draft; writing – review and editing. **Martha M Zarou:** Data curation; formal analysis; investigation; visualization; writing – review and editing. **Zuzana Brabcova:** Data curation; formal analysis; investigation; visualization; writing – review and editing. **Bodhayan Prasad:** Data curation; formal analysis; visualization. **Désirée Zerbst:** Investigation. **Daniele Sarnello:** Investigation. **Eric R Kalkman:** Investigation. **Angela Ianniciello:** Investigation. **Mary T Scott:** Formal analysis. **Karen Dunn:** Investigation. **Engy Shokry:** Methodology. **David Sumpton:** Formal analysis; methodology. **Mhairi Copland:** Resources; writing – review and editing. **Saverio Tardito:** Resources; writing – review and editing. **Johan Vande Voorde:** Formal analysis; writing – review and editing. **Francis Mussai:** Formal analysis; writing – review and editing. **Paul Cheng:** Resources; writing – review and editing. **G Vignir Helgason:** Conceptualization; resources; formal analysis; supervision; funding acquisition; investigation; writing – original draft; project administration; writing – review and editing.

## Disclosure and competing interests statement

MC has received research funding from Cyclacel and Incyte, is/has been an advisory board member for Novartis, Incyte, Jazz Pharmaceuticals, Pfizer and Servier, and has received honoraria from Astellas, Novartis, Incyte, Pfizer and Jazz Pharmaceuticals. All other authors declare that they have no competing interests.

## Supporting information



Expanded View Figures PDF

Table EV1

Table EV2

PDF+

Source Data for Figure 1

Source Data for Figure 2

Source Data for Figure 3

Source Data for Figure 4

Source Data for Figure 5

## Data Availability

The datasets produced in this study has been deposited to Gene Expression Omnibus GSE226887 (https://www.ncbi.nlm.nih.gov/geo/query/acc.cgi?acc=GSE226887).
