# Supplementary material for: Arginine dependency is a therapeutically exploitable vulnerability in chronic myeloid leukaemic stem cells
Source: EMBO Rep. 2023 Jul 25;24(10):e56279. doi: 10.15252/embr.202256279 (PMC10561355; doi:10.15252/embr.202256279)
Supplement: Supplementary file 1 — Expanded View Figures PDF [file EMBR-24-e56279-s002.pdf]

Expanded View Figures

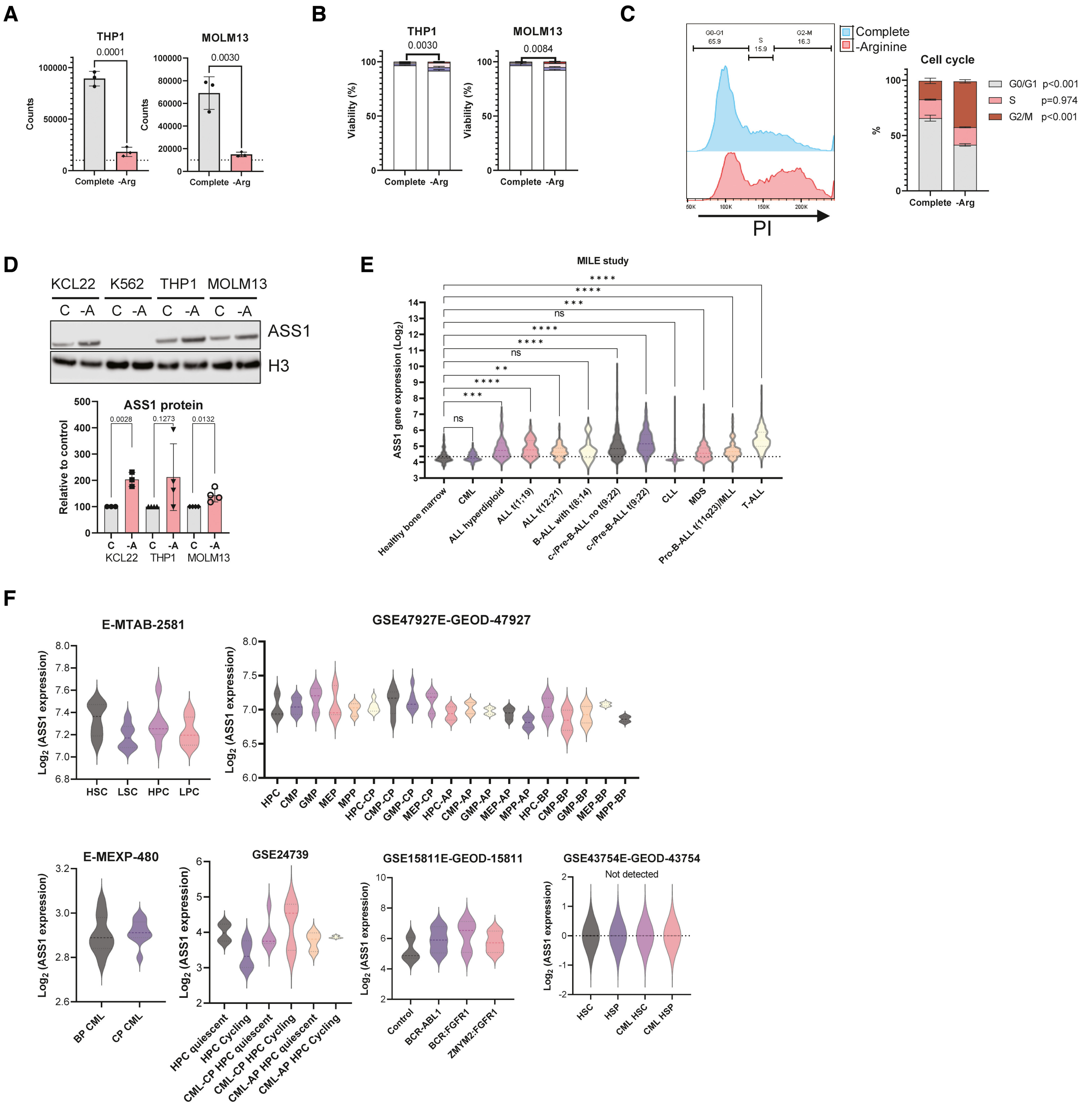

Figure EV1.

Figure EV1. Absence of ASS1 is a feature of CML patient samples.

A Indicated cell lines grown for 72 h in complete medium or medium deficient in arginine and cell number recorded (per well). Cells were seeded at 10,000 cells (dashed line) in 200 μl/well in three replicate plates. Mean, and SEM are plotted.

B Indicated cell lines grown for 72 h in complete medium or medium deficient in arginine and viability measured. Three independent experiments are shown with mean and SEM. Live cells (Annexin V−, 7-AAD−) were analysed.

C Representative plot showing propidium iodide (RNASE-PI) staining after culturing K562 cells with or without arginine for 72 h. Three independent experiments are shown with mean, and SD.

D Indicated cell lines grown for 16 h in complete medium or medium deficient in arginine and ASS1 protein levels were quantified. One representative plot is shown in top panel. Below, 3–4 independent experiments are shown with mean and SEM.

E ASS1 expression from the MILE study. The dotted line revers for average of Healthy Bone Marrow; ** refers to $P < 0.0021$, *** refers to $P < 0.0002$ and ****refers to $P < 0.0001$.

F ASS1 expression in indicated stem-cell enriched datasets. CP: chronic phase, AP: accelerated phase, BP: blast phase. HPC and LPC refer to normal and leukaemic progenitor cells respectively. CMP, GMP, MEP and MPP refer to common myeloid, granulocyte-macrophage, megakaryocyte-erythrocyte, and multipotent progenitors respectively. Markers used in these studies were E-MTAB-2581: CD34, CD38, GSE47927E-GEOD-47927: $CD34^+$, GSE43754E-GEOD-43754: CD34, CD38, ALDH-high, E-MEXP-480: CD34, GSE24739: CD34, Hoechst, GSE15811E-GEOD-15811: CD34 transduced with indicated transgene.

Data information: An unpaired *t*-test was performed for statistical analysis for (A, B and D), a two-way ANOVA with Šíák's multiple comparisons test was performed for (C), Kruskal-Wallis test was performed on (E, F).

Figure EV2. Primary CML samples lack functional urea cycle.

A Schematic showing stable isotope tracing strategy for urea cycle enzymes that are abbreviated as follows; ARG: arginase, OTC: ornithine transcarbamylase, ASS1: arginosuccinate synthase 1, ASL: argininosuccinic acid lyase.

B Results from 48 h tracing of indicated labelled amino acid in CML $CD34^+$ patient samples ($^{13}C_6$ arginine, $^{13}C_5$ ornithine and $^{13}C_6$ citrulline). Isotopologues are indicated in figure legend and *n*-number on bar plots. Data is presented as mean and SEM.

C Diagram showing how generation of arginosuccinate can divert aspartate away from nucleotide synthesis.

D Schematic showing how $^{13}C_5$ glutamine contributes to *de novo* synthesis of pyrimidines by donating three carbons to pyrimidines via aspartate.

E Analysis of $^{13}C_5$ glutamine labelling in *de novo* synthesis of pyrimidines ($n = 3$ normal patient and $n = 6$ CML $CD34^+$ samples for the left panel, $n = 4$ CML $CD34^+$ patient samples for the right panel). An unpaired *t*-test was performed for statistical analysis.

 © 2023 The Authors

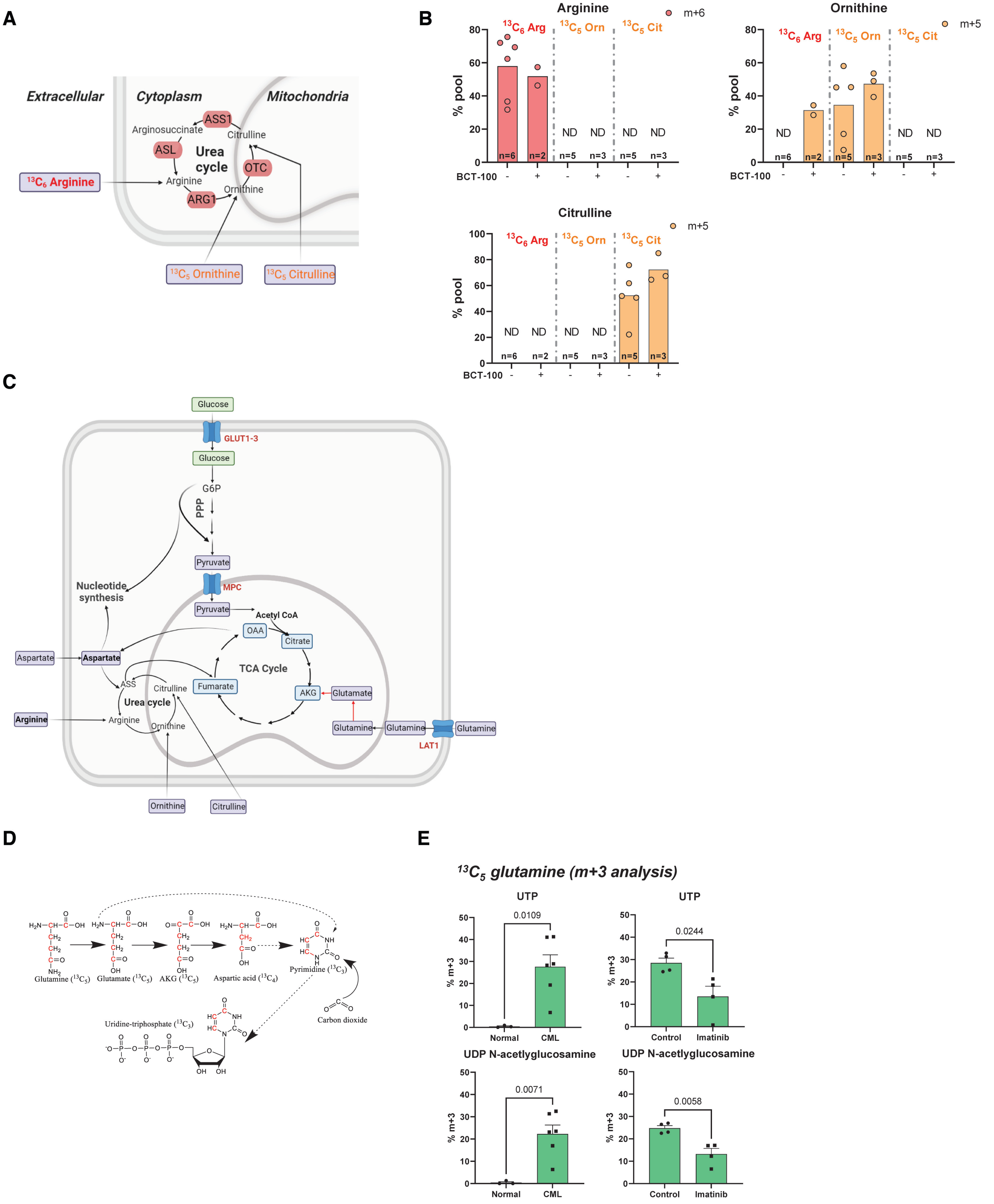
A
Extracellular
Cytoplasm
Mitochondria
ASS1
Arginosuccinate
Citrulline
ASL
Urea cycle
OTC
13C6 Arginine
Arginine
Ornithine
ARG1
13C5 Ornithine
13C5 Citrulline
B
Arginine
m+6
13C6 Arg
13C5 Orn
13C5 Cit
% pool
ND
BCT-100
Ornithine
m+5
Citrulline
C
Glucose
GLUT1-3
G6P
PPP
Pyruvate
MPC
Nucleotide synthesis
Acetyl CoA
OAA
Citrate
Aspartate
TCA Cycle
ASS
Citrulline
Fumarate
AKG
Glutamate
Urea cycle
Arginine
Ornithine
Glutamine
LAT1
D
Glutamine (13C5)
Glutamate (13C5)
AKG (13C5)
Aspartic acid (13C4)
Pyrimidine (13C3)
Carbon dioxide
Uridine-triphosphate (13C3)
E
13C5 glutamine (m+3 analysis)
UTP
% m+3
0.0109
0.0244
Normal
CML
Control
Imatinib
UDP N-acetlyglucosamine
0.0071
0.0058

Figure EV2.

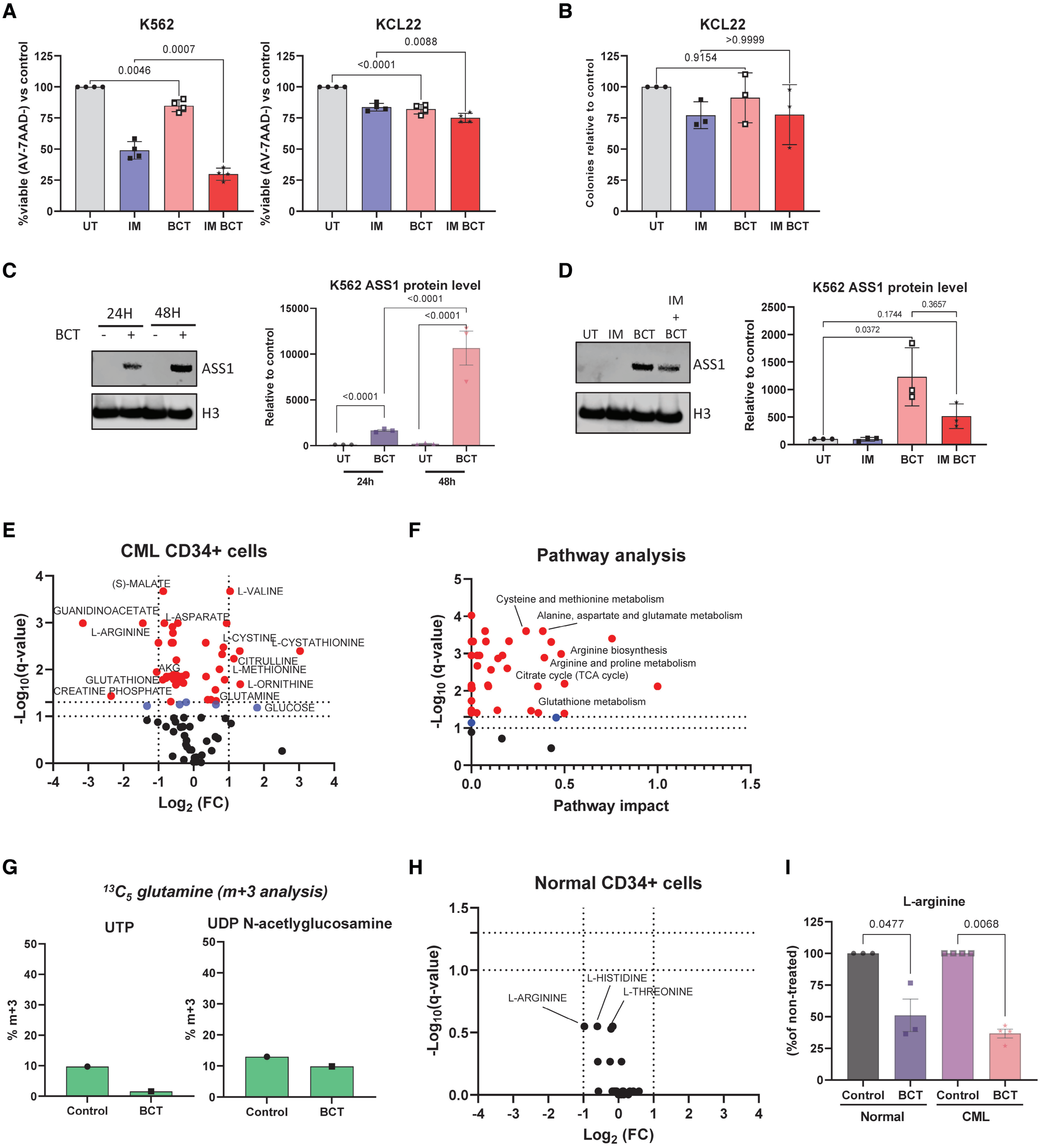

Figure EV3.

 © 2023 The Authors

Figure EV3. ASS1 is required for CML cell lines to escape apoptosis induced by arginine starvation.

- A K562 or KCL22 cells were grown for 72 h in imatinib (600 nM), BCT-100 (1,000 ng/ml) or combination and viability measured. Data from four independent experiments are shown. Mean and SEM are shown.
- B KCL22 cells were treated as in (A) before seeding for CFCs. Data from three independent experiments are shown. Mean and SEM are shown.
- C Western blotting was used to visualise ASS1 protein levels in K562 cells exposed to BCT-100 (1,000 ng/ml) for indicated times. On the left a representative blot is shown. The right panel shows data from three independent experiments. Mean and SEM is plotted.
- D Western blotting was used to visualise ASS1 protein levels in K562 cells as treated in Fig 3A. The left panel shows a representative blot. The right panel shows data from three independent experiments. Mean and SEM is plotted.
- E Volcano plot from LC–MS analysis of CML $CD34^+$ cells (n = 4 patients) treated with BCT-100 (100 ng/ml) for 24 h. Blue denotes q-value < 0.1, red denotes q-value < 0.05.
- F Pathway analysis of (E). Blue denotes q-value < 0.1, red denotes q-value < 0.05 (Benjamini & Hochberg).
- G Analysis of $^{13}C_5$ glutamine labelling in *de novo* synthesis of pyrimidines one CML $CD34^+$ samples that was treated with BCT-100 (100 ng/ml) for 24 h.
- H Volcano plot from LC–MS analysis of normal $CD34^+$ cells (n = 3 biological samples) treated with BCT-100 (100 ng/ml) for 24 h.
- I L-arginine from (E) (4 patient samples) and (H) (n = 3 biological samples). Mean and SEM is plotted.

Data information: An ordinary one-way ANOVA with Tukey's correction for multiple comparisons were performed on data from (A–C) (data from (C) was log-transformed to ensure normality). Kruskal-Wallis test with the Benjamini and Hochberg false discover correction was used to analyse data for (D). Metabolanalyst was used to calculate q-values after mean-centering and R-Log transforming data for (E) and (H). For (F), pathway analysis was conducted using Metabolanalyst after mean-centering and R-Log transforming data. The Globaltest and relative betweenness centrality were used on Homo sapiens KEGG database. A Kruskal-Wallis test was used to analyse data for (I).

Figure EV4. Pharmacological arginine depletion uniqely effects CML patient samples with drastic effects on both control and ASS1 KO cells.

- A Indicated cell lines, normal or CML $CD34^+$ cells were treated for 24 h with BCT-100 (1,000 ng/ml for cell lines and 100 ng/ml for primary samples). ASS1 counts from indicated cell lines (three independent experiments), normal samples (n = 3 biological replicates) and CML patient samples (n = 4 biological replicates) are shown. Mean and SD is plotted.
- B PCA for samples in (A).
- C PCA for primary samples only.
- D Volcano plot showing differentially expressed genes between normal and CML $CD34^+$ cells, red denotes q-value < 0.05, blue denotes q-value < 0.1.
- E Volcano plot showing differentially expressed genes between normal $CD34^+$ vehicle and BCT-100 treated cells, red denotes q-value < 0.05.
- F Venn diagram showing differentially expressed genes from indicated comparisons.
- G Volcano plot showing differentially expressed genes between Control and ASS1 KD K562 cells, red denotes q-value < 0.05, blue denotes q-value < 0.1.
- H Volcano plot showing differentially expressed genes between Control K562 cells treated with BCT-100 or vehicle, red denotes q-value < 0.05, blue denotes q-value < 0.1.
- I Volcano plot showing differentially expressed genes between ASS1 KD K562 cells treated with BCT-100 or vehicle, red denotes q-value < 0.05, blue denotes q-value < 0.1.
- J Downregulated KEGG pathways from GSEA analysis on genes from the intersect of KD-BCT and Control-BCT are shown. Here pi values (computed by multiplying $\log_2$ fold change by $-\log_{10}$ (q-value)) from Control-BCT were used. In red are sets with corrected P-values less than false discovery rate threshold (P < 0.25: dotted line).
- K Downregulated KEGG pathways from GSEA analysis on genes from the intersect of KD-BCT and Control-BCT are shown. Here pi values from KD-BCT were used. In red are sets with corrected P-value less than false discovery rate threshold (P < 0.25: dotted line).
- L The upregulated pathways corresponding to (J) are shown.
- M The upregulated pathways corresponding to (K) are shown. DESEQ2 and GSEA were used as described in methods.

© 2023 The Authors

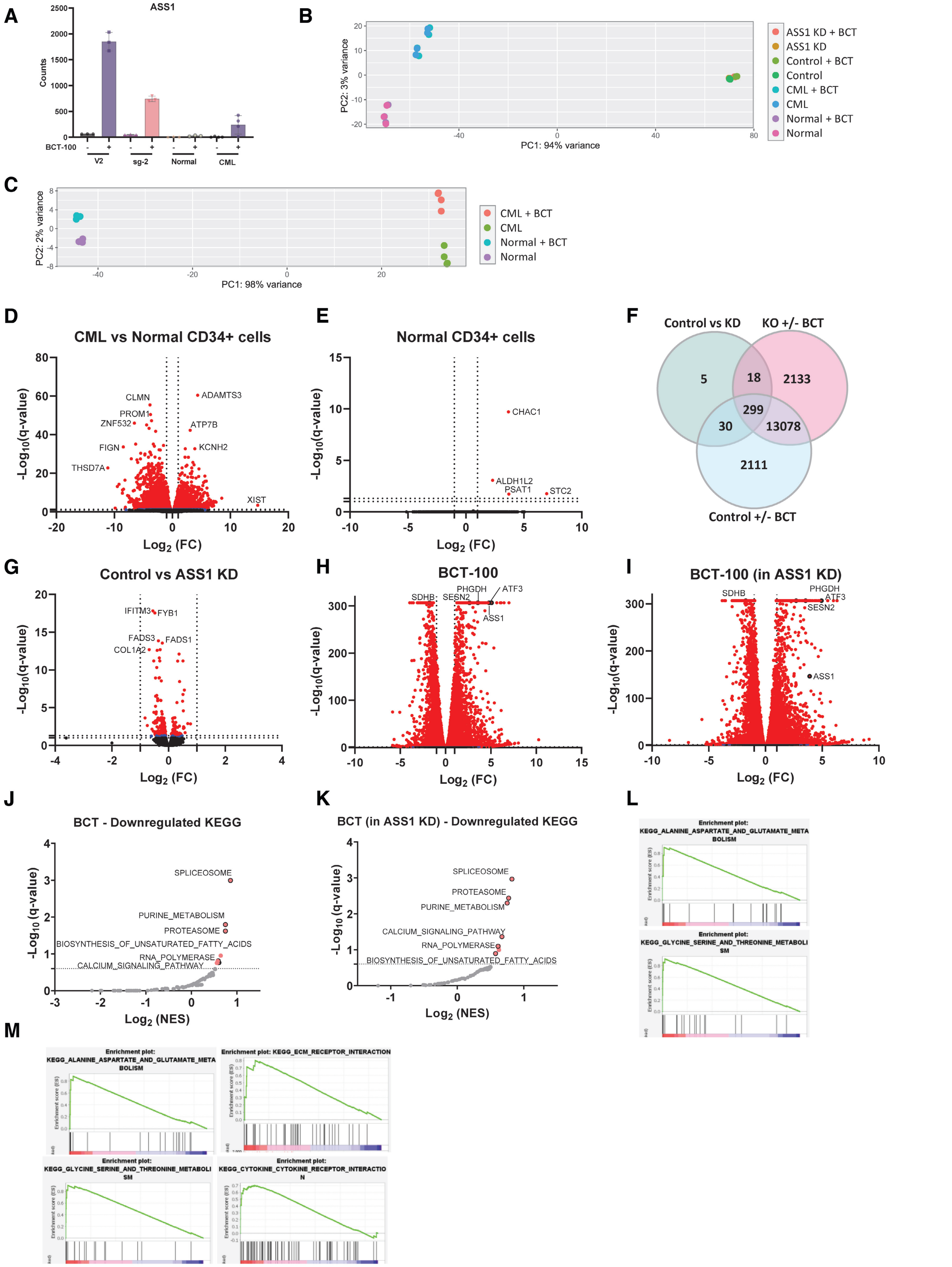

Figure EV4.

© 2023 The Authors

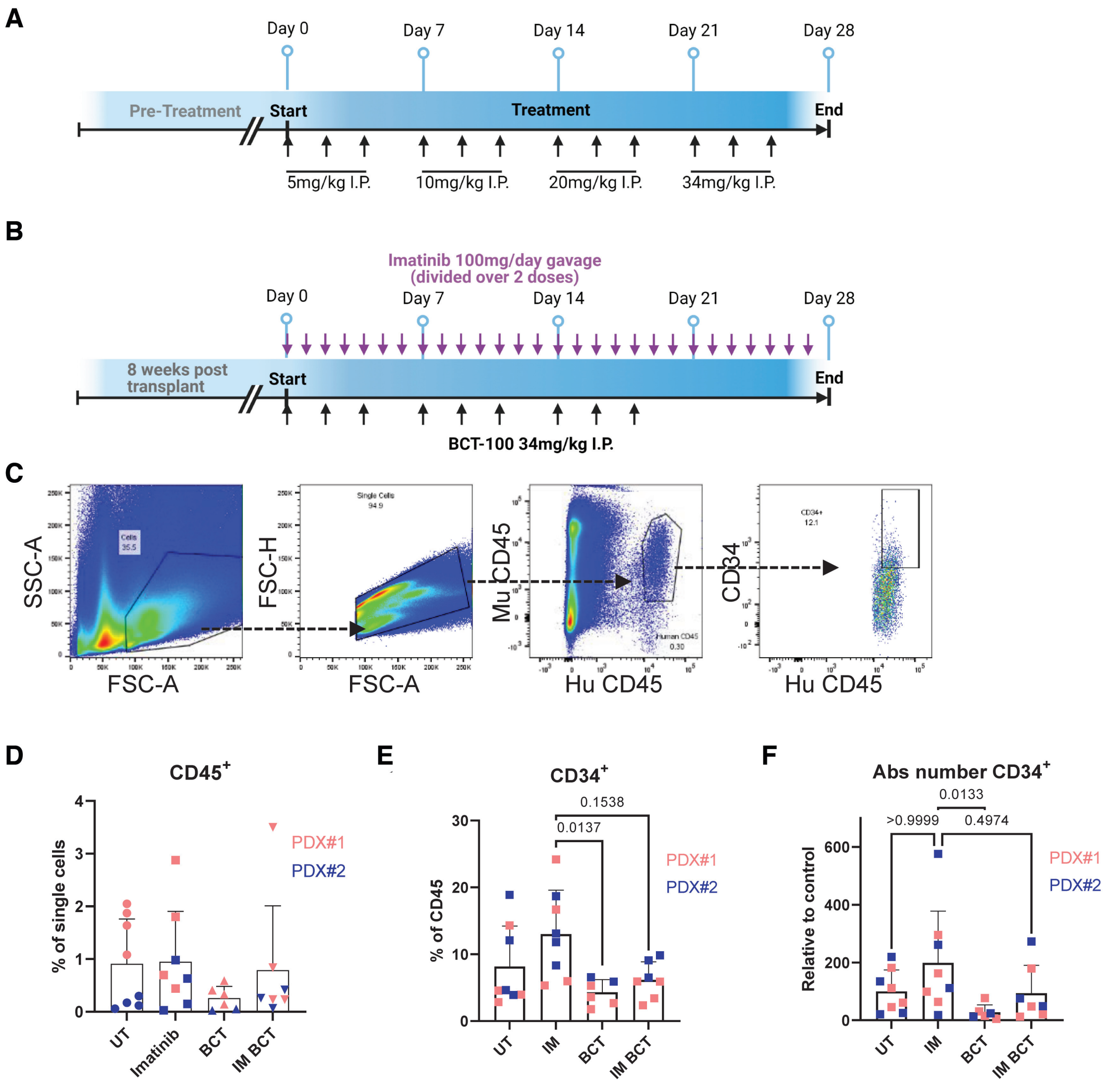

Figure EV5. Pharmacological arginine depletion is human effective in CML LSC xenograft model.

A Dosing strategy for escalation study. Serum samples were taken prior to first dose, each first escalation dose and 24 h after last dose.

B Dosing strategy for PDX experiment.

C Gating strategy is shown for flow cytometry analysis.

D The percentage of $CD45^+$ cells from single cells is shown. Biological replicate data from all mice, average and SD are plotted. Vehicle: $n = 8$ mice, Imatinib: $n = 8$ mice, BCT-100: $n = 6$ mice, Combo: $n = 7$ mice.

E The percentage of $CD34^+$ cells (from CD45) is shown. Average and SD are plotted. Biological replicate data from all mice, average and SD are plotted. Vehicle: $n = 8$ mice, Imatinib: $n = 8$ mice, BCT-100: $n = 6$ mice, Combo: $n = 7$ mice.

F The absolute number of $CD34^+$ cells is shown. Average and SD are plotted. Biological replicate data from all mice, average and SD are plotted. Vehicle: $n = 8$ mice, Imatinib: $n = 8$ mice, BCT-100: $n = 6$ mice, Combo: $n = 7$ mice.

Data information: A Kruskal-Wallis test was used to analyse data for (D–F) with significant changes (adjusted *P*-value, all *P*-values in (D) are > 0.5) or relevant comparisons are shown.
