# Supplementary material for: Arginine dependency is a therapeutically exploitable vulnerability in chronic myeloid leukaemic stem cells
Source: EMBO Rep. 2023 Jul 25;24(10):e56279. doi: 10.15252/embr.202256279 (PMC10561355; doi:10.15252/embr.202256279)
Supplement: Supplementary file 2 — Table EV1 [file EMBR-24-e56279-s009.docx]

**Patient samples used:**

| ID | Sex | Age | BCR-ABL status | Other notes | Appears in |
| --- | --- | --- | --- | --- | --- |
|  | | | | | |
| Normal#1 | M | 66 |  | DLBCL | F1D, F2C+E, F3F+H, EVF3H-I, F4A, EVF4A-E |
| Normal#2 | M | 65 |  | Total hip replacement, Osteoarthritis | F1D |
| Normal#3 | M | 69 |  | Mantle Cell Lymphoma | EVF2E (left panel) |
| Normal#4 | F | 80 |  | Left hip replacement, Osteoarthritis | EVF2E (left panel) |
| Normal#5 | M | 60 |  | DLBCL | EVF2E (left panel) |
| Normal#6 | F | 47 |  | Total hip replacement, Osteoarthritis | F3F+H |
| Normal#7 | M | 70 |  | Left hip replacement | F3H |
| Normal#8 | M | 59 |  | Right hip replacement | F3H |
| Normal#9 | M | 48 |  | Lymphoma | F2C+E, F3F+H, EVF3H-I, F4A, EVF4A-E |
| Normal#10 | M | 70 |  | Total hip replacement, Osteoarthritis | F2E, F3H |
| Normal#11 | M | 67 |  | Lymphoma | F2C, F4A, EVF4A-E |
| Normal#12 | F | 71 |  | Right hip replacement | EVF3H-I |
|  | | | | | |
| CML#1 | M | 61 | BCR-ABL Positive | response to imatinib: BCR-ABL 2.3% at 12 months | F1D, EVF2E (right panel) |
| CML#2 | M | 56 | BCR-ABL Positive | ELN failure: BCR-ABL 0.11% at 12 months, MMR by 18 months: not resistant | F1D, EVF2E (left and right panel) |
| CML#3 | F | 35 | BCR-ABL Positive | Optimal response to imatinib (MR4) | F1D, EVF2B, EVF2E (right panel), F3G, EVF3E-F+H-I |
| CML#4 | F | 52 | BCR-ABL Positive | response imatinib: BCR-ABL <10% at 12 months | F2A-B+D, EVF2B |
| CML#5 | M | 70 | BCR-ABL Positive | no TKI, no molecular monitoring; allogeneic SCT. Cured | EVF2B |
| CML#6 | M | 24 | BCR-ABL Positive | Optimal response to imatinib at 3 months: BCR-ABL 0.07%, -> dasatinib due to side effects | EVF2B |
| CML#7 | M | 58 | BCR-ABL Positive | ELN warning in CP on imatinib | EVF2E (left panel) |
| CML#8 | M | 39 | BCR-ABL Positive | ELN failure Nilotinib at diagnosis -> dasatinib | EVF2E (left panel) |
| CML#9 | M | 61 | BCR-ABL Positive | ELN failure | EVF2E (left panel), F3D-E+G, EVF3E-F+H-I |
| CML#10 | M | 46 | BCR-ABL Positive | ELN failure Imatinib->nilotinib->dasatinib->SCT | EVF2E (left panel) |
| CML#11 | F | 63 | BCR-ABL Positive | Failed imatinib -> dasatinib | EVF2E (left panel) |
| CML#12 | M | 61 | BCR-ABL Positive | Initial optimal response to imatinib, loss of response -> dasatinib; mutation screen negative; in MMR; optimal response. | EVF2E (right panel) |
| CML#13 | M | 47 | BCR-ABL Positive | ELN warning on dasatinib | F3D-E+G |
| CML#14 | M | 39 | BCR-ABL Positive | Failed imatinib (compliance issues) | F3D-E+G |
| CML#15 | F | 60 | BCR-ABL Positive | Suboptimal response to imatinib 400mg, increased to 600mg, then reduced to 400mg, optimal response: BCR-ABL 0.01% | F3D-E+G, EVF3E-F+H-I |
| CML#16 | M | 70 | BCR-ABL Positive | Failed imatinib -> dasatinib, died | F3E+G, F5F-H, EVF5B-F |
| CML#17 | F | 27 | BCR-ABL Positive | ELN warning, on imatinib in MMR | F3G, EVF3E-F+H-I, F4A-E, EVF4A-D |
| CML#18 | F | 61 | BCR-ABL Positive | Failed imatinib -> dasatinib (in DMR) | EVF2B, F4A-E, EVF4A-D |
| CML#19 | M | 33 | BCR-ABL Positive | On Imatinib | EVF2B, EVF3G, F4A-E, EVF4A-D |
| CML#20 | M | 55 | BCR-ABL Positive | Imatinib->dasatinib (Therapy interrupted due to neutropenia intermittently) and responding | EVF3H, F5B, F4A-E, EVF4A-D |

**Table EV1: Patient samples used in this study**

-> refers to next treatment; ELN (European Leukaemia-Net recommendations for the management of CML); DLBCL; Diffuse large B cell lymphoma; E: Extended Data Figure; CP: chronic phase; SCT: stem cell transplantation; MMR: major molecular response, BCR-ABL1<0.1% international scale (IS); MR4: BCR-ABL1 ≤0.01% international scale (IS); MR2: BCR-ABL1 <1% IS
