# Supplementary material for: Arginine dependency is a therapeutically exploitable vulnerability in chronic myeloid leukaemic stem cells
Source: EMBO Rep. 2023 Jul 25;24(10):e56279. doi: 10.15252/embr.202256279 (PMC10561355; doi:10.15252/embr.202256279)
Supplement: Supplementary file 3 — Table EV2 [file EMBR-24-e56279-s007.docx]

| Up | | | |
| --- | --- | --- | --- |
| Metabolite | Fold change | q-value | Component |
| L-CYSTATHIONINE | 8.2 | 0.0 | MI |
| GLUCOSE | 3.5 | 0.1 | P |
| L-ORNITHINE | 2.5 | 0.0 | P |
| L-CYSTINE | 2.5 | 0.0 | P |
| L-METHIONINE | 2.2 | 0.0 | P |
| L-VALINE | 2.1 | 0.0 | P |
| GLYCEROL 3-PHOSPHATE | 1.9 | 0.0 | MI |
| CHOLINE | 1.8 | 0.0 | P |
| L-TRYPTOPHAN | 1.8 | 0.0 | P |
| L-PHENYLALANINE | 1.8 | 0.0 | P |
| CITRULLINE | 1.7 | 0.0 | P |
| L-TYROSINE | 1.6 | 0.0 | P |
| L-LYSINE | 1.6 | 0.1 | P |
| L-SERINE | 1.5 | 0.0 | P |
| L-ASPARAGINE | 1.4 | 0.0 | P |
| GLUTAMINE | 1.3 | 0.0 | P |
| L-THREONINE | 1.3 | 0.0 | P |
| L-ALANINE | 1.3 | 0.0 | P |
| Down | | | |
| GLUTAMATE | 0.9 | 0.1 | P/MI |
| ADENOSINE 5'-DIPHOSPHATE | 0.9 | 0.0 | MI |
| D-(-)-FRUCTOSE | 0.8 | 0.0 | MI |
| NAD | 0.8 | 0.0 | MI |
| S-ADENOSYL-L-METHIONINE (SAM) | 0.8 | 0.1 | MI |
| ACETYL-ASPARTATE (N) | 0.8 | 0.0 | MI |
| PANTOTHENIC ACID | 0.8 | 0.0 | MI |
| TAURINE | 0.7 | 0.0 | P/MI |
| HISTAMINE | 0.7 | 0.0 | MI |
| CYTIDINE 5'-TRIPHOSPHATE | 0.7 | 0.0 | MI |
| GLUTATHIONE | 0.7 | 0.0 | P/MI |
| ATP | 0.7 | 0.0 | MI |
| D-PANTOTHENIC ACID | 0.7 | 0.0 | P |
| AKG | 0.7 | 0.0 | MI |
| PHOSPHOCHOLINE | 0.7 | 0.0 | MI |
| L-CYSTEIC ACID | 0.7 | 0.0 | MI |
| URIDINE 5'-DIPHOSPHOGLUCOSE | 0.7 | 0.0 | MI |
| URIDINE 5'-TRIPHOSPHATE | 0.6 | 0.0 | MI |
| URIDINE MONOPHOSPHATE (UMP) | 0.6 | 0.0 | MI |
| GUANOSINE TRIPHOSPHATE (GTP) | 0.6 | 0.0 | MI |
| PROPIONYL-CARNITINE | 0.6 | 0.0 | MI |
| DIHYDROXYACETONE PHOSPHATE (DHAP) | 0.6 | 0.0 | MI |
| L-ASPARATE | 0.6 | 0.0 | P/MI |
| (S)-MALATE | 0.5 | 0.0 | P/MI |
| S-ADENOSYL-L-HOMOCYSTEINE | 0.5 | 0.0 | MI |
| OROTIDINE | 0.5 | 0.0 | MI |
| PHOSPHOENOLPYRUVATE (PEP) | 0.5 | 0.0 | MI |
| L-2-PHOSPHOGLYCERIC ACID | 0.4 | 0.1 | MI |
| L-ARGININE | 0.4 | 0.0 | P |
| CREATINE PHOSPHATE | 0.2 | 0.0 | MI |
| GUANIDINOACETATE | 0.1 | 0.0 | MI |

**Table EV2: Significantly changed metabolites from Expanded View Figure 3E**

Within Component column, P refers to metabolic input/nutrient that is found in Plasmax and MI refers to metabolic intermediate. Note that Plasmax contains metabolic intermediates that are also present in human plasma.
