# Supplementary figures and images for: Arginine dependency is a therapeutically exploitable vulnerability in chronic myeloid leukaemic stem cells
Source: EMBO Rep. 2023 Jul 25;24(10):e56279. doi: 10.15252/embr.202256279 (PMC10561355; doi:10.15252/embr.202256279)

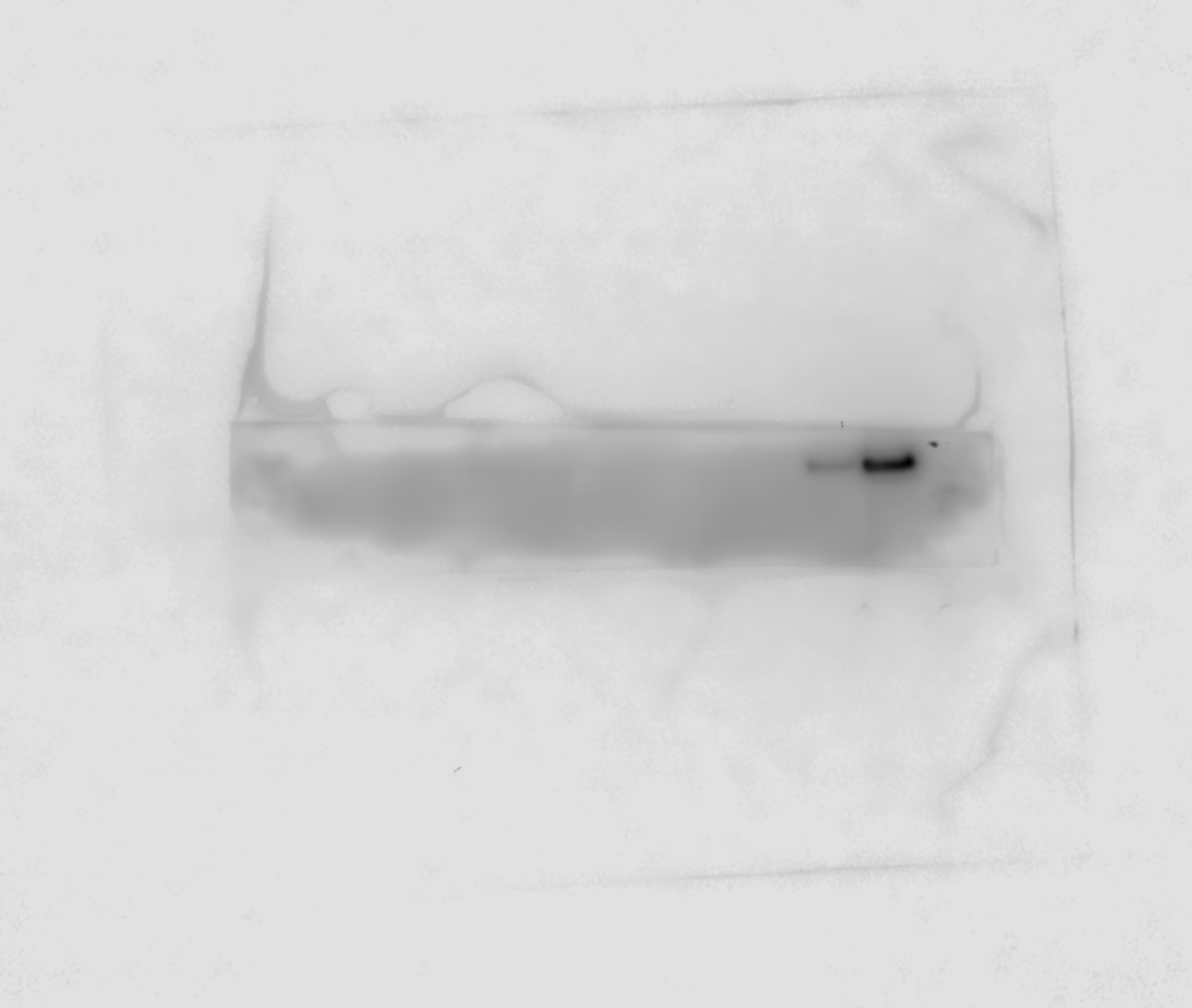

Supplement: Supplementary file 5 — Source Data for Figure 1 [file EMBR-24-e56279-s003.zip › EMBOR-2022-56279V3-Figure_1D_Source_Data-sd.tif]

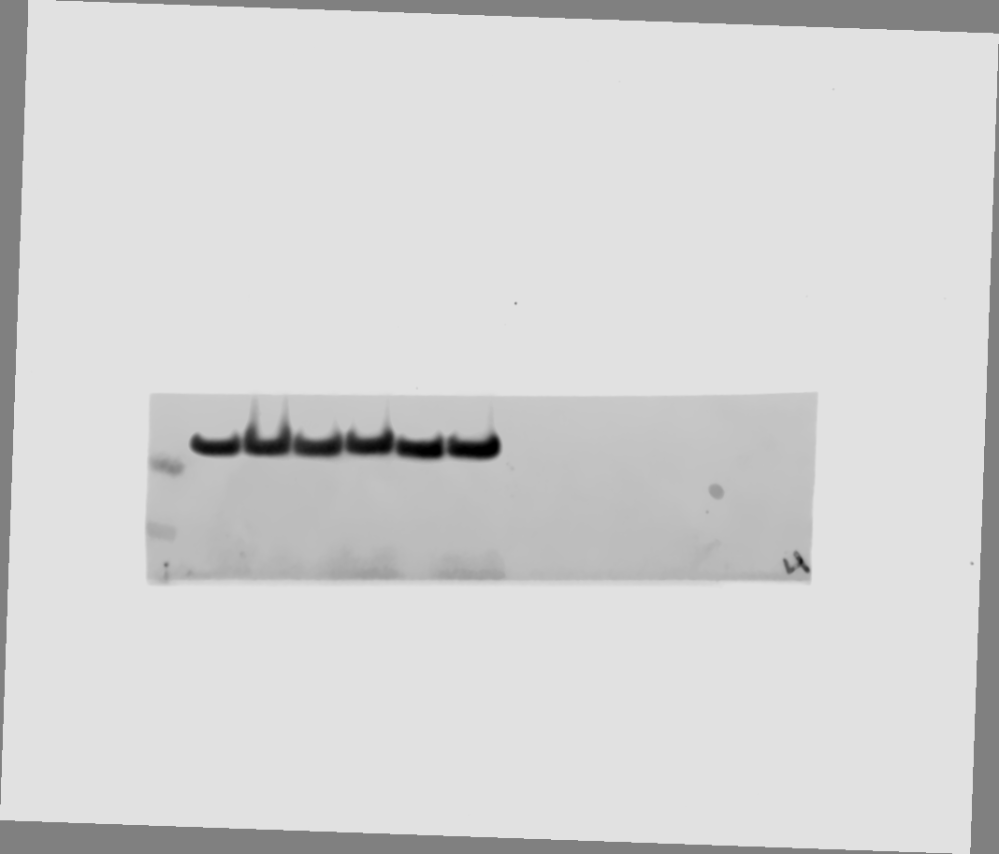

Supplement: Supplementary file 7 — Source Data for Figure 3 [file EMBR-24-e56279-s001.zip › 3B/EMBOR-2022-56279V3-Figure_3B_Source_Data-sd(1).tif]

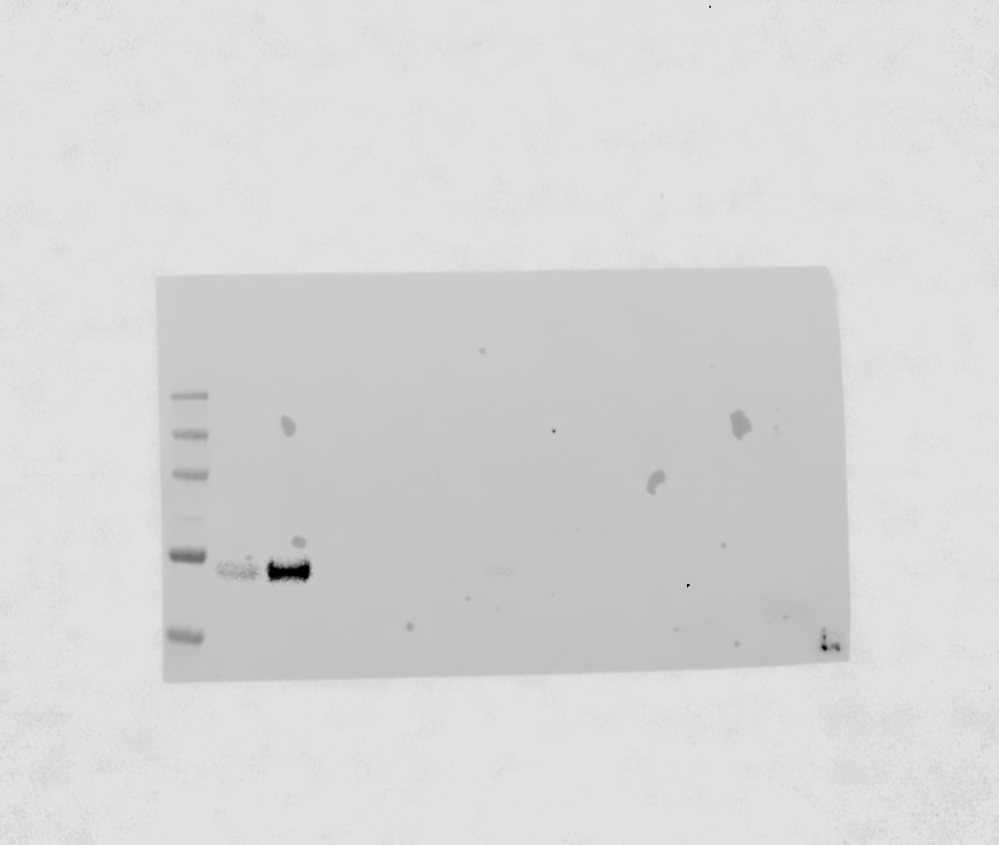

Supplement: Supplementary file 7 — Source Data for Figure 3 [file EMBR-24-e56279-s001.zip › 3B/EMBOR-2022-56279V3-Figure_3B_Source_Data-sd.tif]
