# Supplementary material for: Arginine dependency is a therapeutically exploitable vulnerability in chronic myeloid leukaemic stem cells
Source: EMBO Rep. 2023 Jul 25;24(10):e56279. doi: 10.15252/embr.202256279 (PMC10561355; doi:10.15252/embr.202256279)
Supplement: Supplementary file 10 [file EMBR-24-e56279-s004.pdf]

EMBO reports

Arginine dependency is a therapeutically exploitable vulnerability in chronic myeloid leukaemic stem cells

Kevin Rattigan, Martha Zarou, Zuzana Brabcova, Bodhayan Prasad, Desiree Zerbst, Daniele Sarnello, Eric Kalkman, Angela Ianniciello, Mary Scott, Karen Dunn, Engy Shokry, David Sumpton, Mhairi Copland, Saverio Tardito, Johan Vande Voorde, Francis Mussai, Paul Cheng, and Vignir Helgason
DOI: 10.15252/embr.202256279

Corresponding author(s): Vignir Helgason (Vignir.Helgason@Glasgow.ac.uk)

Review Timeline:

Submission Date:	12th Oct 22
Editorial Decision:	16th Nov 22
Revision Received:	30th Mar 23
Editorial Decision:	5th May 23
Revision Received:	24th Jun 23
Accepted:	3rd Jul 23

Editor: Achim Breiling

Transaction Report:

(Note: With the exception of the correction of typographical or spelling errors that could be a source of ambiguity, letters and reports are not edited. Depending on transfer agreements, referee reports obtained elsewhere may or may not be included in this compilation. Referee reports are anonymous unless the Referee chooses to sign their reports.)

Dear Prof. Helgason,

Thank you for the submission of your research manuscript to EMBO reports. I have already forwarded to you the reports from the three referees that were asked to evaluate your study (which can be found again at the end of this email).

As you have already seen, the referees think that the study is of interest, but all have several concerns, indicating that the study needs a major revision to be considered for publication. Moreover, both referees #1 and #3 indicate novelty issues.

After cross-commenting with the other referees and going through your response (revision plan), I decided to proceed with the manuscript. Please revise the manuscript as indicated, addressing all referee points, adding new data and discussing the novelty concerns. I also think it is important to build upon last concern by referee #1 and provide the experimental data using multiple CML lines throughout.

Given the constructive referee comments, we would like to invite you to revise your manuscript with the understanding that all referee concerns must be addressed in the revised manuscript and in a detailed point-by-point response. Acceptance of your manuscript will depend on a positive outcome of a second round of review. It is EMBO reports policy to allow a single round of revision only and acceptance of the manuscript will therefore depend on the completeness of your responses included in the next, final version of the manuscript.

Revised manuscripts should be submitted within three months of a request for revision. Please contact me to discuss the revision (also by video chat) if you have questions or comments regarding the revision, or should you need additional time.

When submitting your revised manuscript, please also carefully review the instructions that follow below.

PLEASE NOTE THAT upon resubmission revised manuscripts are subjected to an initial quality control prior to exposition to re-review. Upon failure in the initial quality control, the manuscripts are sent back to the authors, which may lead to delays. Frequent reasons for such a failure are the lack of the data availability section (please see below) and the presence of statistics based on n=2 (the authors are then asked to present scatter plots or provide more data points).

When submitting your revised manuscript, we will require:

1) a .docx formatted version of the final manuscript text (including legends for main figures, EV figures and tables), but without the figures included. Figure legends should be compiled at the end of the manuscript text.

2) individual production quality figure files as .eps, .tif, .jpg (one file per figure), of main figures (up to 8) and EV figures. Please upload these as separate, individual files upon re-submission.

The Expanded View format, which will be displayed in the main HTML of the paper in a collapsible format, has replaced the Supplementary information. You can submit up to 5 images as Expanded View. Please follow the nomenclature Figure EV1, Figure EV2 etc. The figure legend for these should be included in the main manuscript document file in a section called Expanded View Figure Legends after the main Figure Legends section. Additional Supplementary material should be supplied as a single pdf file labeled Appendix. The Appendix should have page numbers and needs to include a table of content on the first page (with page numbers) and legends for all content. Please follow the nomenclature Appendix Figure Sx, Appendix Table Sx etc. throughout the text, and also label the figures and tables according to this nomenclature.

For more details, please refer to our guide to authors:
http://www.embopress.org/page/journal/14693178/authorguide#manuscriptpreparation

Please consult our guide for figure preparation:
http://wol-prod-cdn.literatumonline.com/pb-assets/embo-site/EMBOPress_Figure_Guidelines_061115-1561436025777.pdf

See also the guidelines for figure legend preparation:
https://www.embopress.org/page/journal/14693178/authorguide#figureformat

3) a complete author checklist, which you can download from our author guidelines (https://www.embopress.org/page/journal/14693178/authorguide). Please insert page numbers in the checklist to indicate where the requested information can be found in the manuscript. The completed author checklist will also be part of the RPF.

Please also follow our guidelines for the use of living organisms, and the respective reporting guidelines:
http://www.embopress.org/page/journal/14693178/authorguide#livingorganisms

4) that primary datasets produced in this study (e.g. RNA-seq, ChIP-seq, structural and array data) are deposited in an

appropriate public database. If no primary datasets have been deposited, please also state this in a dedicated section (e.g. 'No primary datasets have been generated and deposited'), see below.

See also: http://embor.embopress.org/authorguide#datadeposition

Please remember to provide a reviewer password if the datasets are not yet public.

The accession numbers and database should be listed in a formal "Data Availability" section (placed after Materials & Methods) that follows the model below. This is now mandatory (like the COI statement). Please note that the Data Availability Section is restricted to new primary data that are part of this study. This section is mandatory. As indicated above, if no primary datasets have been deposited, please state this in this section

Data availability

The datasets produced in this study are available in the following databases:

- RNA-Seq data: Gene Expression Omnibus GSE46843 (https://www.ncbi.nlm.nih.gov/geo/query/acc.cgi?acc=GSE46843)
- [data type]: [name of the resource] [accession number/identifier/doi] ([URL or identifiers.org/DATABASE:ACCESSION])

*** Note - All links should resolve to a page where the data can be accessed. ***

Moreover, I have these editorial requests:

6) We now request the publication of original source data with the aim of making primary data more accessible and transparent to the reader. Our source data coordinator will contact you to discuss which figure panels we would need source data for and will also provide you with helpful tips on how to upload and organize the files.

7) Our journal encourages inclusion of *data citations in the reference list* to directly cite datasets that were re-used and obtained from public databases. Data citations in the article text are distinct from normal bibliographical citations and should directly link to the database records from which the data can be accessed. In the main text, data citations are formatted as follows: "Data ref: Smith et al, 2001" or "Data ref: NCBI Sequence Read Archive PRJNA342805, 2017". In the Reference list, data citations must be labeled with "[DATASET]". A data reference must provide the database name, accession number/identifiers and a resolvable link to the landing page from which the data can be accessed at the end of the reference. Further instructions are available at: http://www.embopress.org/page/journal/14693178/authorguide#referencesformat

8) Regarding data quantification and statistics, please make sure that the number "n" for how many independent experiments were performed, their nature (biological versus technical replicates), the bars and error bars (e.g. SEM, SD) and the test used to calculate p-values is indicated in the respective figure legends (also for potential EV figures and all those in the final Appendix). Please also check that all the p-values are explained in the legend, and that these fit to those shown in the figure. Please provide statistical testing where applicable. Please avoid the phrase 'independent experiment', but clearly state if these were biological or technical replicates. Please also indicate (e.g. with n.s.) if testing was performed, but the differences are not significant. In case n=2, please show the data as separate datapoints without error bars and statistics. See also: http://www.embopress.org/page/journal/14693178/authorguide#statisticalanalysis

If n<5, please show single datapoints for diagrams.

9) Please also note our reference format:
http://www.embopress.org/page/journal/14693178/authorguide#referencesformat

10) We updated our journal's competing interests policy in January 2022 and request authors to consider both actual and perceived competing interests. Please review the policy https://www.embopress.org/competing-interests and update your competing interests if necessary. Please name this section 'Disclosure and Competing Interests Statement' and put it after the Acknowledgements section.

11) We now use CRediT to specify the contributions of each author in the journal submission system. CRediT replaces the author contribution section. Please use the free text box to provide more detailed descriptions. See also guide to authors: https://www.embopress.org/page/journal/14693178/authorguide#authorshipguidelines

12) Please order the manuscript sections like this, using these names:
Title page - Abstract - Keywords - Introduction - Results & Discussion - Materials and Methods - Data availability section - Acknowledgements (including funding information) - Disclosure and Competing Interests Statement - References - Figure legends - Expanded View Figure legends

I look forward to seeing a revised version of your manuscript when it is ready. Please let me know if you have questions or

comments regarding the revision.

Please use this link to submit your revision: https://embor.msubmit.net/cgi-bin/main.plex

Yours sincerely,

Achim Breiling
Senior Editor
EMBO Reports

Referee #1:

Rattigan et al, tried to exploit metabolic alterations in a CML setting and have identified arginine depletion as a promising therapeutic strategy. This is an interesting approach and has proven to be effective in solid tumors and hematological malignancies.

Yet it is not discussed at all, that Arginases are already widely used in acute lymphoid leukemia for more than 25 years, hence it is not suprining that it has some effect for CML: This should be at least mentionned in the discussion.

Authors have further shown that BCT100 (a PEGylated human recombinant arginase) reduced viability and clonogenicity of CML cells. They have also demonstrated this data in vivo using mouse models and PDX models. These observations are interesting, but the data presented in the manuscript is not very convincing in the current format and requires further experimentation and significantly improving the overall structure of the manuscript.

Comments:
1. To give a better perspective of the field and current understanding to the reader it would be important to cover recent literature related to Arginine depeletion and its importance in therapy of other cancers/haematological malighancies and associated pathways/ inhibitors in the introduction.
2. 1st sentence of 3rd paragraph is very long and confusing. Please consider rewriting this.
3. Fig 1A: Heat map lacks the required resolution and numbers are blurred.
4. Fig 1B: Assay used is unclear. Authors should consider RNAse-PI method or similar if they want to state that arginine deprivation results in anti-proliferative effect.
5.Supp Fig 1C: The fact that ASS1 upregulation upon arginine deprivation is not evident in the K562 cell line (despite strong H3 band) is not evident. This technical challenge should be addressed.
6. Fig 1D: Unlike in Supp Fig 1C, authors introduced Imatinib treatment and not arginine deprivation and looked for ASS1 expression. The rationale for this is not clear.
7. Fig 2A-2C: Authors should consider experimentation with Non-CML or healthy donor cells. It will be interesting to see normal CD34+ cells react to arginine deprivation.
8.Fig 2B: Its striking that this observation is in contrast to data presented in Fig 1C using K562 and KCl22 where apoptosis was minimal. Authors could consider explaining these differences.
9. Fig 2: With 2 samples/ without statistics authors should rephrase the terms "significant/ substantial differences".
10. Supp Fig 2E: Would have like to see the data in Arg deprived cells.
11. Fig 3A: Would have liked to see ASS1 expression using K562 cell line in the 4 conditions.
12: Fig 3C: Strongest effects were seen in the combination (Imatinib and BCT). Therefore, it would be good to see the CTV curves in the data presented in the Fig 3C.
13. Fig 3B and 3D: Again would have liked to see normal CD34+ cells data.
14. Fig 3E: Representative colony assay image (IM+BCT) donot match with the statistics. This should be corrected.
15. Fig 3E: To avoid confusion authors should stick to normal CD34+ or non-CML.
16. Supp Fig 3: controls (untreated CD34+CML, Normal CD34+, normal CD34+ BCT treatment) are missing. I reckon control samples, at least untreated ones are imp to underscore the alterations!!!
17: Authors should provide brief interpretation of the data outcome in Supp Fig 3 and if they have som overlapping signaling with ASS1. This will be interesting.
18. Fig 4A and 4B: Authors should provide more information on in vivo expt plan (Dosage, treatment freq..etc). Ideally a short scheme??
19. It is interesting that BCT100 targets TKI resistant LSC population. But the effect of BCT on normal CD34+ cells is not evaluated. This makes the interpretation and specificity of BCT100 questionable.
20. Furthermore to substantiate their conclusion " This is the first instance that clinically relevant pharmacological arginine depletion has been demonstrated to target therapy-resistant LSCs, highlighting BCT-100 treatment as a viable strategy to improve current standard of care for CML"
Authors might consider to test effect of BCT 100 in these cells.

21: Authors although used KCL22 in the initial expt's they do not continue with this further. To strengthen their observations, key

expts as Fig 3A should be be reproduced with a 2nd cell line as KCL22.

Referee #2:

Using a medium containing physiological levels of various amino acids, the authors conducted a dropout screen and identified arginine-deprivation as having string anti-proliferative effects in CML and AML cell lines, as well as CML patient samples. Treatment with arginase (BCT-100) significantly reduced CML cell line and primary patient cell growth, but had little effect on normal CD34+ cells. They then performed PD studies in mice and found that BCT-100 treatment reduced arginine levels in the blood as well as bone marrow. Finally, using a xenograft model, they tested the effect of BCT-100 in vivo and found that the growth of CML LSCs was severely reduced.

Overall, this is an interesting paper, the results are clear and the data supports the conclusions. There are a few points that could use clarification:

1. In Figure S2A-B it is not entirely clear what cell types or growth conditions are used for the stable-isotope tracer assay. Presumably, these are CML cells grown in arginine depleted media that don't express ASS1? The cells/growth conditions for this assay should be clarified in the legend.

2. It should be stated more clearly in the figure legend how viability was measured for Figure 3B, otherwise it is difficult to understand what is different in 3B from 3D, as both seem to be measuring colony formation but display different results with the IM/BCT combo.

3. The authors use Plasmax media to better represent normal human blood. One question is whether this is generally representative of blood serum from patients. For example, Crump et al (2021) showed that citrulline supplementation of arginine-media for THP1 (AML cell line) cells essentially stopped working below 100uM (Crump et al, Figure S1H). The Plasmax formulation apparently has citrulline levels of 55uM (Vande Voorde et al, 2019), which is consistent with the inability to support growth of THP-1 cells as shown in Figure S1A of this paper. However, citrulline levels in the plasma from AML patients can be as high as 250uM and displayed an average of 150uM (compared to about 25-50 uM in healthy controls, Crump et al, Figure 1C). At these levels, it seems more likely that citrulline could support the growth of AML cells via ASS1 upregulation. Is anything known about the citrulline levels in the serum of CML patients? The authors should at least acknowledge this point in the paper.

4. Past work from one of the authors has suggested that arginine levels are often low in AML patients, and that this is a sign that they might respond to something like BCT-100 treatment (Mussai et al PMID: 25710880, 2015; Mussai et al PMID: 30485425, 2019; Mussai et al PMID: 23733335, 2013). However, it also possible that some AMLs can tolerate arginine starvation in vivo, as suggested by the observation in other cancers that ASS-positivity is a possible marker for BCT-100 resistance (Chan et al PMID: 33856599, 2021). Thus it could be that low ASS1 expression is a predictor of sensitivity to BCT-100 in patients. Is there any publicly available gene expression data from CML patients available where the authors can explore CML ASS1 expression status?

Referee #3:

In this manuscript, the authors find that CML cell lines and primary CML cells are sensitive to arginine depletion. Arginine withdrawal in cell culture leads to decreased proliferation and apoptosis in CML cell lines as well as in CD34+ CML cells, which represent a fraction of leukemia cells that is enriched for leukemic stem cells. in line with this, arginine depletion by treatment of CML cell lines and primary CML cells with the recombinant arginase BCT-100 reduces clonogenic growth and proliferation, while sparing normal CD34+ cells. Treatment of a CML PDX model with BT-100 in vivo causes a significant decrease in the amount of CD34+CD38- leukemic stem cells.

The results are clear and presented in a logical order and the manuscript is well written and easy to follow. However, while the finding that CML leukemia stem cells are sensitive to arginine depletion is interesting, the relevance of arginine metabolism in leukemia has been demonstrated by several publications before. Thus, the novelty of this work is limited.
In addition, the mechanistic aspects of the observed specificity of the effect on leukemia stem cells over bulk CML cells and normal CD34+ cells are underdeveloped.

Main points:

• While the authors show that the effect of arginine dependency is specific to leukemia stem cells and does not affect bulk CML cells, they also find a reduction of proliferation in established CML cell lines upon arginine depletion. Normal CD34+ cells are not affected by BT-100 treatment. As CML cell lines express ASS1 (At variable levels) but primary CML and normal CD34+ do not, ASS1 expression is likely not responsible for the differential effect. Did the authors investigate expression differences in other

relevant enzymes and/or transporters that could mediate this effect? Any mechanistic insight into the specific dependency of CML leukemia stem cells would greatly advance the significance of the work.
• Does the effect of BT-100 treatment in vivo lead to prolonged survival of treated animals?
• There is a slight discrepancy in ASS1 expression of K562 cells in Figure 1D (ASS1 expression absent) and S1C (low levels of ASS1).

Minor points:

• Figure 1A: What are the values that are shown in this panel? The color scale on the right covers values from 2-8 but the colors do not match the values shown in the heatmap. In addition, the essential amino acids could be labelled in a different color to distinguish them from the ones that are specifically required for the proliferation of CML cells.
• Figure 2A: Data from individual patients should be shown.
• The results described in the last two sentences of the third paragraph of the results and discussion section are not shown.

Dear Dr Breiling,

We thank you for considering the revised version of our manuscript "*Arginine dependency is a therapeutically exploitable vulnerability in chronic myeloid leukaemic stem cells*" for publication in EMBO Reports.

We believe we have now addressed all Reviewer comments and particularly, concerns regarding the novelty of the study as suggested. In this regard we now provide data demonstrating that CML have lower ASS1 expression than most of the leukaemia types, making them truly arginine auxotrophic. We also provide additional metabolomic, transcriptomic and functional data demonstrating that normal CD34+ cells are less affected by arginine depletion compared with patient derived CML cells.

We thank all the reviewers for their constructive comments and believe that by addressing them our manuscript has substantially improved. We have highlighted all the changes in the manuscript accordingly and provide a point-by-point rebuttal to all Reviewer comments. These changes are highlighted in the manuscript and in the rebuttal letter as required.

Point-by-point-response

Referee #1

Rattigan et al, tried to exploit metabolic alterations in a CML setting and have identified arginine depletion as a promising therapeutic strategy. This is an interesting approach and has proven to be effective in solid tumors and hematological malignancies.

Yet it is not discussed at all, that Arginases are already widely used in acute lymphoid leukemia for more than 25 years, hence it is not suprining that it has some effect for CML: This should be at least mentionned in the discussion.

Authors have further shown that BCT100 (a PEGylated human recombinant arginase) reduced viability and clonogenicity of CML cells. They have also demonstrated this data in vivo using mouse models and PDX models. These observations are interesting, but the data presented in the manuscript is not very convincing in the current format and requires further experimentation and significantly improving the overall structure of the manuscript.

Response: We agree with Reviewer 1 that arginine depletion is an interesting approach and are thankful for the constructive feedback.

Regarding novelty concerns from this reviewer, we suspect there has been a confusion over the enzyme we have used. Asparaginases, which can be short lasting (Erwinase; asparaginase Erwinia chrysanthemi) or long lasting (Oncaspar; pegaspargase), deplete asparagine and glutamine, and are currently used to treat acute lymphoid leukemia (ALL). As far as we know, neither arginase nor any other arginine depleting enzyme are currently used to treat ALL.

In response to all reviewer´s comments, we have performed additional experiments that we believe strengthens our message that CML cells are susceptible to arginine depleting therapeutic approach.

Comments:

1. To give a better perspective of the field and current understanding to the reader it would be important to cover recent literature related to Arginine depletion and its importance in therapy of other cancers/haematological malignancies and associated pathways/ inhibitors in the introduction.

Response: The following line has been added to first paragraph (page 3): *"In this context, arginine has emerged as a promising candidate with arginine-degrading enzymes being tested in clinical trials against multiple types of leukaemia, liver cancer, melanoma, prostate cancer, lymphoma, glioblastoma, mesothelioma, sarcoma and lung cancer. However, many of these trials have not been successful with upregulation of arginine recycling enzymes, such as argininosuccinate synthase 1 (ASS1), being reported as an escape mechanism (Zou et al, 2019)."*

2. 1st sentence of 3rd paragraph is very long and confusing. Please consider rewriting this.

This has been broken up into two sentences (page 4): *"Our previous studies on arginine dependency in leukaemic blast cells showed pronounced effect, although these experiments were done in media without urea cycle intermediates, thereby preventing potential rescue via the urea cycle. Whether therapy resistant LSCs are auxotrophic for non-essential amino acids in the presence of their circulatory precursors is unknown."*

3. Fig 1A: Heat map lacks the required resolution and numbers are blurred.

Response: This has now been addressed.

4. Fig 1B: Assay used is unclear. Authors should consider RNAse-PI method or similar if they want to state that arginine deprivation results in anti-proliferative effect.

Response: We have clarified in legend that this is cell counts with initial seeding numbers indicated with dashed line. RNAse-PI method has been used as suggested in new Expanded View Figure 1C.

Relevant text has been added on page 4: *"In K562 cells, arginine deprivation caused the expected cell cycle disruption with an accumulation of cells in G2/M phase (Expanded View Figure 1C) (Alexandrou et al, 2018)."*

5.Supp Fig 1C: The fact that ASS1 upregulation upon arginine deprivation is not evident in the K562 cell line (despite strong H3 band) is not evident. This technical challenge should be addressed.

Response: To visualise ASS1 protein in low-expressing K562 cells requires longer exposure time (which leads to overexposure of bands in the other ASS1 medium/high expressing cell lines). However, to address this, we have performed a time-course and demonstrate that even in K562 cells, ASS1 is upregulated following BCT-100 treatment as shown in Expanded View Figures 3C and 3D. We have also performed RNA sequencing on K562 cells following BCT-100 treatment which confirmed an increase at the transcript level (Expanded View Figure 4A).

Relevant text has been added:

Page 7: *"We subsequently examined ASS1 levels in BCT-100-treated K562 cells. BCT-100 caused a time-dependent increase in ASS1 levels (Expanded View Figure 3C) while this was partially reduced in the presence of imatinib, although this did not reach statistical significance (Expanded View Figure 3D)."*

Page 8: *"To further investigate the cellular response to arginine depletion we performed RNA sequencing (RNA-seq) on the engineered K562 cells as well as primary normal and CML CD34+ cells, in the absence or presence of BCT-100. Despite visible reduction at the protein levels, we observed an increase in ASS1 mRNA levels in BCT-100-treated KD K562 cells, albeit less than in control cells. Notably, the increase in ASS1 following BCT-100 treatment in CML CD34+ cells was less compared to K562 KD cells (Expanded View Figure 4A)."*

6. Fig 1D: Unlike in Supp Fig 1C, authors introduced Imatinib treatment and not arginine deprivation and looked for ASS1 expression. The rationale for this is not clear.

Response: Although ASS1 was not detectable in normal CD34+ cells, we did speculate whether BCR-ABL1 kinase activity lead to suppression of ASS1 in CML CD34+ cells.

We have now added relevant text on page 5: *"It is unlikely that BCR-ABL1 is responsible for ASS1 suppression, as ASS1 was also not detectable by Western blotting in imatinib-treated CD34+ CML cells (Figure 1D)."*

7. Fig 2A-2C: Authors should consider experimentation with Non-CML or healthy donor cells. It will be interesting to see normal CD34+ cells react to arginine deprivation.

Response: We agree with this point and have performed additional experiments to test the effect of arginine depletion on viability and colony formation potential of normal CD34+ cells. This revealed that arginine restriction had less effect on normal CD34+ cells compared to CML CD34+ cells. New data are presented in Figures 2C and 2E (should be compared to 2B and 2D).

Relevant text has been added on page 6: "*Importantly, we saw less effect on the viability and clonogenicity in normal CD34+ cells (Figures 2C and 2E)*."

8.Fig 2B: Its striking that this observation is in contrast to data presented in Fig 1C using K562 and KCl22 where apoptosis was minimal. Authors could consider explaining these differences.

Response: We agree that the difference between primary CML cells and cell lines in intriguing. As mentioned above (Point 5), we present additional data demonstrating that ASS1 is upregulated at the protein and transcript level following arginine depletion in low-expressing K562 cells (Expanded View Figures 3C, 3D and 4A). We also demonstrate that ASS1 knock-down sensitises K562 cells to BCT-100 (new Figures 3B and 3C). The fact ASS1 expression is even lower in CML CD34+ cells compared to K562 cells (Expanded View Figures 4A) may

explain the increased sensitivity of primary cells to arginine depletion (although we accept that the precise mechanism for these differences warrants further investigation).

9. Fig 2: With 2 samples/ without statistics authors should rephrase the terms "“significant/ substantial differences"”.

Response: We thank the reviewer for pointing this out and have performed additional experiments, increased n-numbers and provided statistical analysis for relevant figures.

10. Supp Fig 2E: Would have like to see the data in Arg deprived cells.

Response: We do not expect that glutamine metabolism would specifically be affected in arginine depleted cells given that steady state levels of most metabolic intermediates were reduced (Expanded View Figure 3E and Supplementary Table 2). Nevertheless, we have performed additional experiment on one patient sample with results presented in new Expanded View Figure 3G.

Relevant text has been added on page 8: *“Subsequent analysis of 13C5 glutamine into pyrimidines in BCT-100 treated CML samples showed that this was decreased (Expanded View Figure 3G). However, as steady state levels of most metabolic intermediates were decreased, we cannot preclude that the incorporation of other carbon sources that produce aspartate are also reduced.”*

11. Fig 3A: Would have liked to see ASS1 expression using K562 cell line in the 4 conditions.

Response: These data are now presented in new Expanded View Figure 3D. We found the results quite interesting but are unsure if the modest reduction in ASS1 levels following imatinib treatment would sensitise the cells to arginine deprivation.

Relevant text has been added on page 7: *“BCT-100 caused a time-dependent increase in ASS1 levels (Expanded View Figure 3C) while this was partially reduced in the presence of imatinib, although this did not reach statistical significance (Expanded View Figure 3D).”*

12: Fig 3C: Strongest effects were seen in the combination (Imatinib and BCT). Therefore, it would be good to see the CTV curves in the data presented in the Fig 3C.

Response: We agree that this is informative, with new analysis demonstrating that BCT-100 treatment has more anti-proliferative effect compared with imatinib treatment (new Figure 3D).

Relevant text has been added on page 7: *“Similar to arginine starvation, BCT-100 treatment potently blocked proliferation of CML CD34+ cells to a greater extent than imatinib single treatment (Figure 3D).”*

13. Fig 3B and 3D: Again, would have liked to see normal CD34+ cells data.

Response: In addition to new experiments in response to Point 7 (arginine deprivation), we treated additional normal CD34+ cells with BCT-100 and assessed the effect on viability and colony formation potential (new Figure 3F, and Figure 3H with increased sample size). These experiments confirmed that normal CD34+ cells are less sensitive to arginine restriction than CML counterparts.

Relevant text has been added on page 7: *“No significant effect of BCT-100 treatment was observed on normal CD34+ cells, in contrast to cells treated with the with omacetaxine*

mepesuccinate (OMA), an inhibitor of protein biosynthesis, used on occasion for advanced phases of CML (Figure 3F)."

14. Fig 3E: Representative colony assay image (IM+BCT) do not match with the statistics. This should be corrected.

Response: While there is visible patient sample variability, we have aimed to select a representative picture for visual purposes only. Note, the data is normalised relative to the control. Counts were recorded manually as many colonies were not detectable during scanning due to size or lack of colour (prior to imaging, cells were incubated with MTT reagent). If the reviewer insists, we can remove all assay images in the manuscript and present only the graphs with statistical analysis.

15. Fig 3E: To avoid confusion authors should stick to normal CD34+ or non-CML.

Response: Thank-you, we have now corrected this and stuck to "normal CD34+" for clarity.

16. Supp Fig 3: controls (untreated CD34+CML, Normal CD34+, normal CD34+ BCT treatment) are missing. I reckon control samples; at least untreated ones are imp to underscore the alterations!!!

Response: We have now performed additional experiments with normal CD34+ cells (Expanded View Figures 3H and 3I). Note, we have previously characterised the differences between normal and CML CD34+ cells (Kuntz *et al,* Nature Medicine 2017, 23, pages 1234–1240).

Relevant text has been added on page 8: *"Finally, we performed additional experiments on normal CD34+ cells. While the changes here failed to reach significance following multivariate analysis (Expanded View Figure 3H), when examining L-arginine levels, we confirmed a consistent decrease in both normal and CML datasets (Expanded View Figure I)."*

17: Authors should provide brief interpretation of the data outcome in Supp Fig 3 and if they have some overlapping signalling with ASS1. This will be interesting.

Response: We now provide additional findings in relation to Expanded View Figure 3 and present results regarding ASS1 levels in these experiments (Figure 4C and Expanded View Figure 4A present transcript levels for ASS1 in normal and CML cells following BCT-100 treatment). Regarding metabolomic experiments (Expanded View Figures 3E-I), the key results provide evidence of BCT-100 activity, with reduced arginine levels and increased ornithine levels, both in normal and CML CD34+ cells. Interestingly, citrulline levels are also increased in CML CD34+ cells (Expanded View Figure 3E), although the biological significance warrants further investigation.

Relevant new text has been added on pages 8-9: *"The majority of differentially expressed genes in BCT-treated CML were upregulated, including ASS1 and arginine transporter SLC7A3 (CAT3) (Figure 4C). Other upregulated genes included those related to translation (ATF3, EIF1) and serine metabolism (PSAT1, ALDH1L2, PHGDH, MTHFD2 and SHMT2). In agreement with this, gene set enrichment analysis (GSEA) showed that upregulated pathways included one carbon metabolism and nitrogen metabolism (Figure 4D-E). These findings agree with previous studies demonstrating that arginine deprivation leads to upregulation of serine*

biosynthesis in ASS1 deficient breast, melanoma, and sarcoma cell lines (Cheng et al, 2018; Kremer et al, 2017).”

18. Fig 4A and 4B: Authors should provide more information on in vivo expt plan (Dosage, treatment freq..etc). Ideally a short scheme??

Response: We now provide a schematic demonstration of drug escalation and treatment plan for the relevant figures (new Expanded View Figures 5A and 4B).

19. It is interesting that BCT100 targets TKI resistant LSC population. But the effect of BCT on normal CD34+ cells is not evaluated. This makes the interpretation and specificity of BCT100 questionable.

Response: We have made a huge effort in addressing this point experimentally and performed metabolomic and transcriptomic analysis of normal CD34+ cells following BCT-100 treatment, in addition to functional survival experiments. Overall, our results (new main Figures 3 and 4, and Expanded View Figure 3 and 4) suggest that normal CD34+ cells are less affected by BCT-100, which is consistent with relatively good safety profile in clinical trial. We believe these data add novelty and significantly increase the potential impact of our findings.

Relevant text has been added:

Page 7: *“No significant effect of BCT-100 treatment was observed on normal CD34+ cells, in contrast to cells treated with the with omacetaxine mepesuccinate (OMA), an inhibitor of protein biosynthesis, used on occasion for advanced phases of CML (Figure 3F).”*

Page 8: *“Finally, we performed additional experiments on normal CD34+ cells. While the changes here failed to reach significance following multivariate analysis (Expanded View Figure 3H), when examining L-arginine levels, we confirmed a consistent decrease in both normal and CML datasets (Expanded View Figure 3I).*

Page 8: *“Differential expression analysis confirmed that most significantly differentially expressed genes were between CML and normal, with BCT-100 altering levels of 82 genes in CML cells and only 4 in normal cells (Figure 4A-B, Expanded View Figure 4D-E).”*

20. Furthermore to substantiate their conclusion "This is the first instance that clinically relevant pharmacological arginine depletion has been demonstrated to target therapy-resistant LSCs, highlighting BCT-100 treatment as a viable strategy to improve current standard of care for CML" Authors might consider to test effect of BCT 100 in these cells.

Response: We have clarified in the text that CML LSCs are intrinsically therapy-resistant (primary resistance). We do not have access to samples from patients who have developed “acquired TKI resistance” (i.e., BCR-ABL kinase domain mutation) if that is what the reviewer is suggesting.

Relevant text has been added on page 10: *“Notably this effect was most evident in the BM-located LSC population that is intrinsically TKI-resistant (Hamilton et al., 2012).”*

21: Authors although used KCL22 in the initial expt’’s they do not continue with this further. To strengthen their observations, key expts as Fig 3A should be be reproduced with a 2nd cell line as KCL22.

Response: We have now performed key experiments with the KCL22 cell line as suggested (new data presented in Expanded View Figures 3A and 3B). KCL22 cells (express high ASS1 levels) were not affected by BCT-100 treatment, possibly due to having higher ASS1 levels. Unfortunately, we were unable to generate ASS1 knockdown KCL22 cells, possible due to the additional chromosomal abnormalities existing in this cell line.

Relevant text has been added on page 7: *"BCT-100 significantly reduced the clonogenicity of ASS1-low K562 cells, with a further reduction observed in the presence of imatinib (Figure 3A). These reductions were absent in ASS1-high KCL22 cells (Expanded View Figure 3B)."*

Referee #2

Using a medium containing physiological levels of various amino acids, the authors conducted a dropout screen and identified arginine-deprivation as having string anti-proliferative effects in CML and AML cell lines, as well as CML patient samples. Treatment with arginase (BCT-100) significantly reduced CML cell line and primary patient cell growth but had little effect on normal CD34+ cells. They then performed PD studies in mice and found that BCT-100 treatment reduced arginine levels in the blood as well as bone marrow. Finally, using a xenograft model, they tested the effect of BCT-100 in vivo and found that the growth of CML LSCs was severely reduced. Overall, this is an interesting paper, the results are clear and the data supports the conclusions. There are a few points that could use clarification:

Response: We thank this reviewer for encouraging comments and for describing our work as interesting and clearly presented.

1. In Figure S2A-B it is not entirely clear what cell types or growth conditions are used for the stable-isotope tracer assay. Presumably, these are CML cells grown in arginine depleted media that don''t express ASS1? The cells/growth conditions for this assay should be clarified in the legend.

Response: For clarification, these experiments were performed in complete medium (containing arginine). We performed separate experiments using 3 separate tracers ($^{13}C_6$ arginine; $^{13}C_5$ ornithine; $^{13}C_5$ citrulline). Incorporation of labelled carbons into the 3 amino acids was then presented (indicated amino acid is labelled). We have now reorganised the data with each plot containing a given metabolite and tracer indicated on x-axis. We have also performed additional experiments following BCT-100 treatment. Reassuringly, we see no evidence of urea cycle activity other than the expected BCT-100 (human arginase 1) mediated conversion of $^{13}C_6$ arginine to $^{13}C_5$ ornithine.

This has been clarified in figure legend and relevant text has been added on page 5: *"As expected, treatment with recombinant human arginase BCT-100 (catalyses conversion of arginine to ornithine) increased labelling in ornithine from labelled arginine (middle graph, second bar) (Cheng et al, 2007). However, there were no additional changes when arginine was depleted using BCT-100, further suggesting absence of urea cycle activity."*

2. It should be stated more clearly in the figure legend how viability was measured for Figure 3B, otherwise it is difficult to understand what is different in 3B from 3D, as both seem to be measuring colony formation but display different results with the IM/BCT combo.

Response: For clarification, Figure 3B presents viability (now Figure 3E), assessed following staining with annexin V (AV) and 7-amino-actinomycin D (7-AAD), with viable cells defined as negative for AV and 7-AAD (AV-/7AAD-). This has now been added to the y-axis for clarification. Data are presented relative to control. Figure 3D (now 3G) presents colony numbers, relative to control.

3. The authors use Plasmax media to better represent normal human blood. One question is whether this is generally representative of blood serum from patients. For example, Crump et al (2021) showed that citrulline supplementation of arginine- media for THP1 (AML cell line) cells essentially stopped working below 100uM (Crump et al, Figure S1H). The Plasmax formulation apparently has citrulline levels of 55uM (Vande Voorde et al, 2019), which is

consistent with the inability to support growth of THP-1 cells as shown in Figure S1A of this paper. However, citrulline levels in the plasma from AML patients can be as high as 250uM and displayed an average of 150uM (compared to about 25-50 uM in healthy controls, Crump et al, Figure 1C). At these levels, it seems more likely that citrulline could support the growth of AML cells via ASS1 upregulation. Is anything known about the citrulline levels in the serum of CML patients? The authors should at least acknowledge this point in the paper.

Response: We thanks the reviewer for these valuable points. We have now referenced the work in Crump *et al* (2021) and added the relevant text on page 6: *"Crump et al showed that elevated levels (150 µM) of plasma citrulline found in AML patients can support the growth of AML cell lines via ASS1 upregulation following arginine depravation (Crump et al., 2021). However, citrulline levels in CML patients remains to be determined."*

While we don't have access to patient data to comment of citrulline levels in plasma from CML patients, using surplus patient samples, we tested whether 250 µM citrulline rescues the effect of BCT-100 on colony forming potential of CML CD34+ cells. In our hands, citrulline supplementation does not rescue the effect of BCT-100 in our system (see Rebuttal Figure 1). To avoid disrupting the flow of the paper, we have not added this data to our manuscript. However, if this reviewer insists, we can add the data as Expanded View Figure in our revised version.

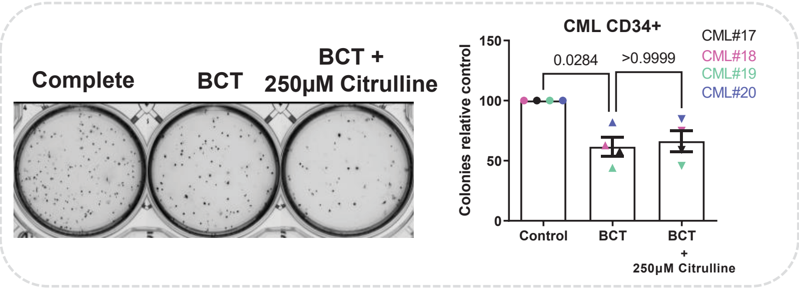

Rebuttal Figure 1

4. Past work from one of the authors has suggested that arginine levels are often low in AML patients, and that this is a sign that they might respond to something like BCT-100 treatment (Mussai et al PMID: 25710880, 2015; Mussai et al PMID: 30485425, 2019; Mussai et al PMID: 23733335, 2013). However, it also possible that some AMLs can tolerate arginine starvation in vivo, as suggested by the observation in other cancers that ASS-positivity is a possible marker for BCT-100 resistance (Chan et al PMID: 33856599, 2021). Thus it could be that low ASS1 expression is a predictor of sensitivity to BCT-100 in patients. Is there any publicly available gene expression data from CML patients available where the authors can explore CML ASS1 expression status?

Response: We thank the reviewer for this valuable suggestion. We have now interrogated publicly available CML datasets as suggested. These include datasets comparing CML with AML subclassed, myelodysplastic syndrome (MDS) and control normal bone marrow from the Microarray Innovations in Leukaemia (MILE) study and other datasets using various stem cell markers. These data, now presented in Figure 1E and Expanded View Figure 1E-F, reveal that CML cells have consistently low ASS1 expression when compared with AML and most other leukaemia types. Furthermore, there was no significant difference in expression between

CML stem cells (CD34+CD38- or quiescent) and progenitors, accelerated phase or blast phase populations. Additionally, our RNA sequencing experiment confirms low mRNA ASS1 expression in primary cells compare with CML cell lines (Expanded View Figure 4A).

Relevant text has been added on page 5: *"Given the undetectable ASS1 protein levels in primary normal and CML cells, we next examined ASS1 gene expression from the Microarray Innovations in Leukemia (MILE) study (Bagger et al, 2016; Haferlach et al, 2010) (Figure 1E and Expanded View Figure 1E). Here we found that CML and normal bone marrow (BM) cells have low ASS1 mRNA expression compared to other leukaemia types. As ASS1 expression levels are known determinant of sensitivity to arginine deprivation, we subsequently examined ASS1 expression in indicated progenitor and stem-cell enriched datasets that include chronic phase, accelerated phase and blast phase samples (Expanded View Figure 1F). While these studies use different markers such as CD34, CD38 (Cramer-Morales et al, 2013; Scott et al, 2016; Zheng et al, 2006), Hoechst (Gerber et al, 2013), or CD34+ cells transduced with indicated BCR-ABL1 transgenes (Agerstam et al, 2010), no significant differences in ASS1 levels were detected. This data would predict a consistent response to arginine deprivation across CML subtypes."*

Page 8: *"To further investigate the cellular response to arginine depletion we performed RNA sequencing (RNA-seq) on the engineered K562 cells as well as primary normal and CML CD34+ cells, in the absence or presence of BCT-100. Despite visible reduction at the protein levels, we observed an increase in ASS1 mRNA levels in BCT-100-treated KD K562 cells, albeit less than in control cells. Notably, the increase in ASS1 following BCT-100 treatment in CML CD34+ cells was less compared to K562 KD cells (Expanded View Figure 4A)."*

Referee #3

In this manuscript, the authors find that CML cell lines and primary CML cells are sensitive to arginine depletion. Arginine withdrawal in cell culture leads to decreased proliferation and apoptosis in CML cell lines as well as in CD34+ CML cells, which represent a fraction of leukemia cells that is enriched for leukemic stem cells. In line with this, arginine depletion by treatment of CML cell lines and primary CML cells with the recombinant arginase BCT-100 reduces clonogenic growth and proliferation, while sparing normal CD34+ cells. Treatment of a CML PDX model with BT-100 in vivo causes a significant decrease in the amount of CD34+CD38- leukemic stem cells.

The results are clear and presented in a logical order and the manuscript is well written and easy to follow. However, while the finding that CML leukemia stem cells are sensitive to arginine depletion is interesting, the relevance of arginine metabolism in leukemia has been demonstrated by several publications before. Thus, the novelty of this work is limited.

In addition, the mechanistic aspects of the observed specificity of the effect on leukemia stem cells over bulk CML cells and normal CD34+ cells are underdeveloped.

Response: We thank this reviewer for encouraging comments and for describing our work as clear, logical, and well written. To increase the novelty and translational significance of our work we provide additional metabolomic, transcriptomic and functional data demonstrating that normal CD34+ cells are less affected by arginine depletion compared with patient derived CML cells. These data are presented in new main Figures 2-4 and Expanded View Figures 3-4 and are consistent with relatively good safety profile of BCT-100 in clinical trials (see also comment to Reviewer 1, point 19). We have also interrogated publicly available transcriptomic datasets and provide novel data demonstrating that CML have low ASS1 expression compared with other leukaemia types, making them truly arginine auxotrophic. These data are now presented in Figure 1E and Expanded View Figure 1E-F (see also comment to Reviewer 2, point 4).

Relevant text has been added:

Page 5: *"Given the undetectable ASS1 protein levels in primary normal and CML cells, we next examined ASS1 gene expression from the Microarray Innovations in Leukemia (MILE) study (Bagger et al, 2016; Haferlach et al, 2010) (Figure 1E and Expanded View Figure 1E). Here we found that CML and normal bone marrow (BM) cells have low ASS1 mRNA expression compared to other leukaemia types. As ASS1 expression levels are known determinant of sensitivity to arginine deprivation, we subsequently examined ASS1 expression in indicated progenitor and stem-cell enriched datasets that include chronic phase, accelerated phase and blast phase samples (Expanded View Figure 1F). While these studies use different markers such as CD34, CD38 (Cramer-Morales et al, 2013; Scott et al, 2016; Zheng et al, 2006), Hoechst (Gerber et al, 2013), or CD34+ cells transduced with indicated BCR-ABL1 transgenes (Agerstam et al, 2010), no significant differences in ASS1 levels were detected. This data would predict a consistent response to arginine deprivation across CML subtypes."*

Page 6: *"Importantly, we saw less effect on the viability and clonogenicity in normal CD34+ cells (Figures 2C and 2E)."*

Page 7: *"No significant effect of BCT-100 treatment was observed on normal CD34+ cells, in contrast to cells treated with the with omacetaxine mepesuccinate (OMA), an inhibitor of protein biosynthesis, used on occasion for advanced phases of CML (Figure 3F)."*

Page 8: *"Finally, we performed additional experiments on normal CD34+ cells. While the changes here failed to reach significance following multivariate analysis (Expanded View Figure 3H), when examining L-arginine levels, we confirmed a consistent decrease in both normal and CML datasets (Expanded View Figure 3I)."*

Page 8: *"Differential expression analysis confirmed that most significantly differentially expressed genes were between CML and normal, with BCT-100 altering levels of 82 genes in CML cells and only 4 in normal cells (Figure 4A-B, Expanded View Figure 4D-E)."*

Main points:

While the authors show that the effect of arginine dependency is specific to leukemia stem cells and does not affect bulk CML cells, they also find a reduction of proliferation in established CML cell lines upon arginine depletion. Normal CD34+ cells are not affected by BT-100 treatment. As CML cell lines express ASS1 (At variable levels) but primary CML and normal CD34+ do not, ASS1 expression is likely not responsible for the differential effect.

Response: While we think ASS1 contributes to BCT-100 resistance in CML cell lines (new knockdown data in Figure 3B-C) we agree with the reviewer's comments that ASS1 is not solely responsible to the differential effect in primary CML and normal cells. As mentioned above, our metabolomic and transcriptomic data demonstrate that primary CML cells respond differently to BCT-100 when compared to normal counterparts. It is possible that the increased metabolic activity in CML cells (when compared with normal) makes them more vulnerable to arginine depletion and might to some degree provide and an explanation for the difference in sensitivity to BCT-100.

1 Did the authors investigate expression differences in other relevant enzymes and/or transporters that could mediate this effect? Any mechanistic insight into the specific dependency of CML leukemia stem cells would greatly advance the significance of the work.

Response: As mentioned above we now show that ASS1 knockdown sensitises K562 cells to BCT-100 induced apoptosis, confirming a role for ASS1 in resistance to arginine depletion (Figure 3B-C). This combined with experiments showing no evidence of urea cycle activity in primary CML samples strongly suggests that ASS1 plays a role (interestingly, the level of ASS1 upregulation is less in CML CD34+ cells than in ASS1 knockdown K562 cell). Additionally, our RNA-seq experiment on normal and CML CD34+ cells revealed an upregulation of both ASS1 and the arginine transporter SLC7A3 (CAT3) in CML CD34+ cells. However, we don't fully understand why normal cells are not as affected when they lack ASS1 and have mentioned this in the updated manuscripts.

Relevant text has been added on page 6: *"It is important to note that changes to metabolic demands or proliferation that can alter the requirement of arginine to normal cells, such as T-cells, is context dependent such as reported during immune therapy (Mussai et al, 2019). As such, it is possible that normal cells would become similarly sensitive during haemopoietic expansion following myeloablative treatment."*

Relevant text has been added on pages 8-9: *"The majority of differentially expressed genes in BCT-treated CML were upregulated, including ASS1 and arginine transporter SLC7A3 (CAT3) (Figure 4C). Other upregulated genes included those related to translation (ATF3, EIF1) and serine metabolism (PSAT1, ALDH1L2, PHGDH, MTHFD2 and SHMT2). In agreement with this, gene set enrichment analysis (GSEA) showed that upregulated pathways included one carbon metabolism and nitrogen metabolism (Figure 4D-E). These findings agree with previous studies demonstrating that arginine deprivation leads to upregulation of serine biosynthesis in ASS1 deficient breast, melanoma, and sarcoma cell lines (Cheng et al, 2018; Kremer et al, 2017). In contrast to the primary samples, BCT-100 caused the majority of changes in the K562 cell line, irrespective of ASS1 levels (Expanded View Figure 4F-I) with both control and KD having similar pathways deregulated (Expanded View Figure 4J-M). Further studies will be needed to define the role of upregulated serine biosynthesis in arginine deprived CML cells and if this upregulation is evident in patients with leukaemia or other ASS1-defiecent cancers."*

2 Does the effect of BT-100 treatment in vivo lead to prolonged survival of treated animals?

Response: Given the relative low engraftment of primary chronic phase CML samples to mouse bone marrow, the CML PDX model is solely used to measure human CML stem cell survival but does not support measurement of mouse survival. The following line, and relevant references, have been added on page 9 for clarification: *"While mice in this model do not develop lethal disease, the model is the gold standard for assessment of human CML LSC survival as the duration of engraftment ensures that cells present at end of treatment are LSCs or LSC-derived (Abraham et al, 2016; Ianniciello et al, 2021; Kuntz et al., 2017)."*

3. There is a slight discrepancy in ASS1 expression of K562 cells in Figure 1D (ASS1 expression absent) and S1C (low levels of ASS1).

Response: To visualise ASS1 protein in low-expressing K562 cells requires longer exposure time (which leads to overexposure of bands in the other ASS1 medium/high expressing cell lines). However, to address this, we have performed a time-course and demonstrate that even in K562 cells, ASS1 is upregulated following BCT-100 treatment as shown in Expanded View Figures 3C and 3D. We have also performed RNA sequencing on K562 cells following BCT-100 treatment which confirmed an increase at the transcript level (Expanded View Figure 4A).

Relevant text has been added:

Page 7: *"We subsequently examined ASS1 levels in BCT-100-treated K562 cells. BCT-100 caused a time-dependent increase in ASS1 levels (Expanded View Figure 3C) while this was partially reduced in the presence of imatinib, although this did not reach statistical significance (Expanded View Figure 3D)."*

Page 8: *"To further investigate the cellular response to arginine depletion we performed RNA sequencing (RNA-seq) on the engineered K562 cells as well as primary normal and CML CD34+ cells, in the absence or presence of BCT-100. Despite visible reduction at the protein levels, we observed an increase in ASS1 mRNA levels in BCT-100-treated KD K562 cells, albeit less than in control cells. Notably, the increase in ASS1 following BCT-100 treatment in CML CD34+ cells was less compared to K562 KD cells (Expanded View Figure 4A)."*

Minor points:

1. Figure 1A: What are the values that are shown in this panel? The color scale on the right cover's values from 2-8 but the colors do not match the values shown in the heatmap. In addition, the essential amino acids could be labelled in a different colors to distinguish them from the ones that are specifically required for the proliferation of CML cells.

Response: Colour key has been addressed and values explained. Essential amino acids are now coloured in red.

2. Figure 2A: Data from individual patients should be shown.

Response: We have performed additional experiments; this is now n=3 individual patient samples (with individual patient data shown).

3. The results described in the last two sentences of the third paragraph of the results and discussion section are not shown.

Response: This is now in new Supplemental Table 2.

1st Revision - Editorial Decision 5th May 2023

Dear Prof. Helgason,

Thank you for the submission of your revised manuscript to our editorial offices. I have now received the reports from the three referees that I asked to re-evaluate your study, you will find below. As you will see, the referees now fully support the publication of your study in EMBO reports.

Before proceeding with formal acceptance, I have these editorial requests I ask you to address in a final revised manuscript:

- Please provide the abstract written in present tense.

- Please provide individual production quality figure files as .eps, .tif, .jpg (one file per figure), of the main and EV figures. Please upload these as separate, individual files upon re-submission. Please make sure that the EV figures are named and are call out as 'Figure EVx'.

- The two supplementary tables should also be uploaded individually in word or excel and called Table EV# using the file type 'Expanded View'. Please remove these from the main manuscript text fiel.

- We now use CRediT to specify the contributions of each author in the journal submission system. CRediT replaces the author contribution section. Please use the free text box to provide more detailed descriptions and remove the author contributions section from the manuscript text file. See also guide to authors:
https://www.embopress.org/page/journal/14693178/authorguide#authorshipguidelines

- We updated our journal's competing interests policy in January 2022 and request authors to consider both actual and perceived competing interests. Please review the policy https://www.embopress.org/competing-interests and update your competing interests if necessary. Please name this section 'Disclosure and Competing Interests Statement' and put it after the Acknowledgements section.

- Please remove the referee token from the data availability section and make sure that the data is public latest when the manuscript is published online.

- Please order the manuscript sections like this (using these names as headings):
Title page - Abstract - Keywords - Introduction - Results & Discussion - Materials and Methods - Data availability section - Acknowledgements - Disclosure and Competing Interests Statement - References - Figure legends - Expanded View Figure Legends

- Regarding data quantification and statistics, please make sure that the number "n" for how many independent experiments were performed, their nature (biological versus technical replicates), the bars and error bars (e.g. SEM, SD) and the test used to calculate p-values is indicated in the respective figure legends (also for potential EV figures and all those in the final Appendix). Please also check that all the p-values are explained in the legend, and that these fit to those shown in the figure. Please provide statistical testing where applicable. Please avoid the phrase 'independent experiment', but clearly state if these were biological or technical replicates. Please also indicate (e.g. with n.s.) if testing was performed, but the differences are not significant. In case n=2 please show the data as separate datapoints or bars without error bars and statistics. See also:
http://www.embopress.org/page/journal/14693178/authorguide#statisticalanalysis

If n<5, please show single datapoints for diagrams.

- We now request the publication of original source data with the aim of making primary data more accessible and transparent to the reader. Our source data coordinator will contact you to discuss which figure panels we would need source data for and will also provide you with helpful tips on how to upload and organize the files.

- Finally, please find attached a word file of the manuscript text (provided by our publisher) with changes we ask you to include in your final manuscript text and comments. Please use the attached file as basis for further revisions and provide your final manuscript file with track changes, in order that we can see any modifications done.

In addition, I would need from you:
- a short, two-sentence summary of the manuscript (not more than 35 words).
- two to four short bullet points highlighting the key findings of your study (two lines each).
- a schematic summary figure as separate file that provides a sketch of the major findings (not a data image) in jpeg or tiff format (with the exact width of 550 pixels and a height of not more than 400 pixels) that can be used as a visual synopsis on our website.

I look forward to seeing the final revised version of your manuscript when it is ready. Please let me know if you have questions

regarding the revision.

Please use this link to submit your revision: https://embor.msubmit.net/cgi-bin/main.plex

Achim Breiling
Senior Editor
EMBO Reports

Referee #1:

The authors have responded adequately to the different concern raised.

Referee #2:

The authors have addressed my concerns to my satisfaction. In particular, they have better explained two figure panels that I found unclear. In addition, they have changed the emphasis of the paper. This was done in part by including new analysis showing that ASS1 expression is particularly low in CML patients, by better explaining how their CML results fit into the greater context of cancer treatment, and by indicating that citrulline upregulation could potentially be a mechanism for inducing resistance to arginine depletion (especially in AML cells, but apparently not in CML).

For me, what is novel here is the complete lack of ASS1 expression in the CML samples, making CML potentially true arginine auxotrophs. It still remains to be seen if CML as an entire group lacks ASS1 expression, and if there is any potential relapse due to ASS1 upregulation in BCT-100 treated patients in the future. That said, this is an important and novel study, and I think this work will have great interest for the field.

Referee #3:

In the revised version of this manuscript, the authors have added a significant amount of new data and analyses that contribute to strengthening their conclusions. In particular, the new ASS1 knockout data in Figure 3 provide some mechanistic proof that ASS1 contributes to BCT-100 sensitivity in CML cells.
While some points could not be fully clarified, the authors have responded to all my questions with outstanding clarity and scientific rigor. In that regard, it is important to note that despite the new insights that are provided by this work, some open questions remain. This has been clearly stated in the revised version of the manuscript.

The work can be accepted for publication.

Decision letter

- Please provide the abstract written in present tense.
This has now been done.

- Please provide individual production quality figure files as .eps, .tif, .jpg (one file per figure), of the main and EV figures. Please upload these as separate, individual files upon re-submission. Please make sure that the EV figures are named and are call out as 'Figure EVx'.
High resolution .tif files have now been generated for both main and EV figures. In text Figure EVx is now used.

- The two supplementary tables should also be uploaded individually in word or excel and called Table EV# using the file type 'Expanded View'. Please remove these from the main manuscript text field.
This has now been done, the table legends are within new separate files, is this ok or do the legends need to be in main document.

- We now use CRediT to specify the contributions of each author in the journal submission system. CRediT replaces the author contribution section. Please use the free text box to provide more detailed descriptions and remove the author contributions section from the manuscript text file. See also guide to authors:
https://www.embopress.org/page/journal/14693178/authorguide#authorshipguidelines
- We updated our journal's competing interests policy in January 2022 and request authors to consider both actual and perceived competing interests. Please review the policy https://www.embopress.org/competing-interests and update your competing interests if necessary. Please name this section 'Disclosure and Competing Interests Statement' and put it after the Acknowledgements section.
This has now been done.

- Please remove the referee token from the data availability section and make sure that the data is public latest when the manuscript is published online.
This has now been done.

- Please order the manuscript sections like this (using these names as headings):
Title page - Abstract - Keywords - Introduction - Results & Discussion - Materials and Methods - Data availability section - Acknowledgements - Disclosure and Competing Interests Statement - References - Figure legends - Expanded View Figure Legends
This has now been done.

- Regarding data quantification and statistics, please make sure that the number "n" for how many independent experiments were performed, their nature (biological versus technical replicates), the bars and error bars (e.g. SEM, SD) and the test used to calculate p-values is indicated in the respective figure legends (also for potential EV figures and all those in the final Appendix). Please also check that all the p-values are explained in the legend, and that these fit to those shown in the figure. Please provide statistical testing where applicable. Please avoid the phrase 'independent experiment', but clearly state if these were biological or technical replicates. Please also indicate (e.g. with n.s.) if testing was performed, but the differences are not significant. In case n=2 please show the data as separate datapoints or bars without error bars and statistics. See also:
http://www.embopress.org/page/journal/14693178/authorguide#statisticalanalysis
In interest of space it isn't always possible to show all p-values. Following line has been added to page 10: 'Indicated p-values are plotted, if not shown for a given comparison, result was non-

significant (p>0.05).'

If n<5, please show single datapoints for diagrams.

- We now request the publication of original source data with the aim of making primary data more accessible and transparent to the reader. Our source data coordinator will contact you to discuss which figure panels we would need source data for and will also provide you with helpful tips on how to upload and organize the files.

Awaiting correspondence for source data coordinator.

- Finally, please find attached a word file of the manuscript text (provided by our publisher) with changes we ask you to include in your final manuscript text and comments. Please use the attached file as basis for further revisions and provide your final manuscript file with track changes, in order that we can see any modifications done.

This has been, done with changes coloured in red.

In addition, I would need from you:

- a short, two-sentence summary of the manuscript (not more than 35 words).

A complete lack of urea cycle functioning is a feature of primary CML patient samples. This makes them sensitive to arginine deprivation *in vitro* and *in vivo*.

- two to four short bullet points highlighting the key findings of your study (two lines each).

1. CML patient samples lack activity of all urea cycle enzymes
2. Reduced levels of key urea cycle enzyme ASS1 is conserved across all CML subtypes and leukaemic cell type hierarchy
3. Presence of citrulline is unable to rescue effects of arginine deprivation in CML patient samples unlike cell lines which rapidly upregulate ASS1
4. CML patient samples have unique transcriptomic response to arginine deprivation compared to normal cells

- a schematic summary figure as separate file that provides a sketch of the major findings (not a data image) in jpeg or tiff format (with the exact width of 550 pixels and a height of not more than 400 pixels) that can be used as a visual synopsis on our website.

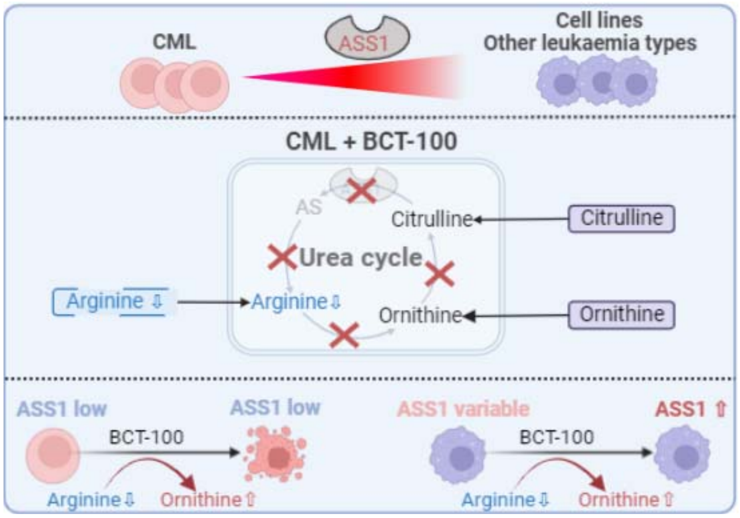

Prof. Vignir Helgason
University of Glasgow
School of Cancer Sciences
Garscube Estate
Garscube G61 1QH
United Kingdom

Dear Prof. Helgason,

I am very pleased to accept your manuscript for publication in the next available issue of EMBO reports. Thank you for your contribution to our journal.

At the end of this email I include important information about how to proceed. Please ensure that you take the time to read the information and complete and return the necessary forms to allow us to publish your manuscript as quickly as possible.

As part of the EMBO publication's Transparent Editorial Process, EMBO reports publishes online a Review Process File to accompany accepted manuscripts. As you are aware, this File will be published in conjunction with your paper and will include the referee reports, your point-by-point response and all pertinent correspondence relating to the manuscript.

If you do NOT want this File to be published, please inform the editorial office within 2 days, if you have not done so already, otherwise the File will be published by default [contact: emboreports@embo.org]. If you do opt out, the Review Process File link will point to the following statement: "No Review Process File is available with this article, as the authors have chosen not to make the review process public in this case."

Should you be planning a Press Release on your article, please get in contact with emboreports@wiley.com as early as possible, in order to coordinate publication and release dates.

Thank you again for your contribution to EMBO reports and congratulations on a successful publication. Please consider us again in the future for your most exciting work.

Yours sincerely,

Achim Breiling
Senior Editor
EMBO Reports

****

THINGS TO DO NOW:

Please note that you will be contacted by Wiley Author Services to complete licensing and payment information. The required 'Page Charges Authorization Form' is available here: https://www.embopress.org/pb-assets/embo-site/er_apc.pdf - please download and complete the form and return to embopressproduction@wiley.com

EMBO Press participates in many Publish and Read agreements that allow authors to publish Open Access with reduced/no publication charges. Check your eligibility: https://authorservices.wiley.com/author-resources/Journal-Authors/open-access/affiliation-policies-payments/index.html

You will receive proofs by e-mail approximately 2-3 weeks after all relevant files have been sent to our Production Office; you should return your corrections within 2 days of receiving the proofs.

Please inform us if there is likely to be any difficulty in reaching you at the above address at that time. Failure to meet our deadlines may result in a delay of publication, or publication without your corrections.

All further communications concerning your paper should quote reference number EMBOR-2022-56279V3 and be addressed to emboreports@wiley.com.

Should you be planning a Press Release on your article, please get in contact with emboreports@wiley.com as early as

possible, in order to coordinate publication and release dates.

EMBO Press Author Checklist

Corresponding Author Name: Vignir Helgason	
Journal Submitted to: EMBO Reports	
Manuscript Number: EMBOR-2022-56279V1	

USEFUL LINKS FOR COMPLETING THIS FORM
The EMBO Journal - Author Guidelines
EMBO Reports - Author Guidelines
Molecular Systems Biology - Author Guidelines
EMBO Molecular Medicine - Author Guidelines

Reporting Checklist for Life Science Articles (updated January 2022)
This checklist is adapted from Materials Design Analysis Reporting (MDAR) Checklist for Authors. MDAR establishes a minimum set of requirements in transparent reporting in the life sciences (see Statement of Task: 10.31222/osf.io/9sm4x). Please follow the journal's guidelines in preparing your manuscript.
Please note that a copy of this checklist will be published alongside your article.

Abridged guidelines for figures
1. Data
The data shown in figures should satisfy the following conditions:
- the data were obtained and processed according to the field's best practice and are presented to reflect the results of the experiments in an accurate and unbiased manner.
- ideally, figure panels should include only measurements that are directly comparable to each other and obtained with the same assay.
- plots include clearly labeled error bars for independent experiments and sample sizes. Unless justified, error bars should not be shown for technical replicates.
- if n<5, the individual data points from each experiment should be plotted. Any statistical test employed should be justified.
- Source Data should be included to report the data underlying figures according to the guidelines set out in the authorship guidelines on Data Presentation.

2. Captions
Each figure caption should contain the following information, for each panel where they are relevant:
- a specification of the experimental system investigated (eg cell line, species name).
- the assay(s) and method(s) used to carry out the reported observations and measurements.
- an explicit mention of the biological and chemical entity(ies) that are being measured.
- an explicit mention of the biological and chemical entity(ies) that are altered/varied/perturbed in a controlled manner.
- the exact sample size (n) for each experimental group/condition, given as a number, not a range;
- a description of the sample collection allowing the reader to understand whether the samples represent technical or biological replicates (including how many animals, litters, cultures, etc.).
- a statement of how many times the experiment shown was independently replicated in the laboratory.
- definitions of statistical methods and measures:
  - common tests, such as t-test (please specify whether paired vs. unpaired), simple χ2 tests, Wilcoxon and Mann-Whitney tests, can be unambiguously identified by name only, but more complex techniques should be described in the methods section;
  - are tests one-sided or two-sided?
  - are there adjustments for multiple comparisons?
  - exact statistical test results, e.g., P values = x but not P values < x;
  - definition of 'center values' as median or average;
  - definition of error bars as s.d. or s.e.m.

Please complete ALL of the questions below.
Select "Not Applicable" only when the requested information is not relevant for your study.

Materials

Newly Created Materials	Information included in the manuscript?	In which section is the information available? (Reagents and Tools Table, Materials and Methods, Figures, Data Availability Section)
New materials and reagents need to be available; do any restrictions apply?	Yes	BCT-100 was provided under MTA, all other materials and reagents are available

Antibodies	Information included in the manuscript?	In which section is the information available? (Reagents and Tools Table, Materials and Methods, Figures, Data Availability Section)
For **antibodies** provide the following information:<br>- Commercial antibodies: RRID (if possible) or supplier name, catalogue number and or/clone number<br>- Non-commercial: RRID or citation	Yes	RRIDS are provided in Material and Methods

DNA and RNA sequences	Information included in the manuscript?	In which section is the information available? (Reagents and Tools Table, Materials and Methods, Figures, Data Availability Section)
**Short novel DNA or RNA including primers, probes**: provide the sequences.	Yes	Sequences are provided

Cell materials	Information included in the manuscript?	In which section is the information available? (Reagents and Tools Table, Materials and Methods, Figures, Data Availability Section)
**Cell lines:** Provide species information, strain. Provide accession number in repository **OR** supplier name, catalog number, clone number, and/**OR** RRID.	Yes	Described in Material and Methods, RRID's given
**Primary cultures:** Provide species, strain, sex of origin, genetic modification status.	Yes	This information is in Supplemental Table 1
Report if the cell lines were recently **authenticated** (e.g., by STR profiling) and tested for mycoplasma contamination.	Yes	Cell lines were authenticated prior to studies and cultures checked monthly for mycoplasma

Experimental animals	Information included in the manuscript?	In which section is the information available? (Reagents and Tools Table, Materials and Methods, Figures, Data Availability Section)
**Laboratory animals or Model organisms:** Provide species, strain, sex, age, genetic modification status. Provide accession number in repository **OR** supplier name, catalog number, clone number, **OR** RRID.	Yes	This is described in Material and Methods
**Animal observed in or captured from the field:** Provide species, sex, and age where possible.	Not Applicable	N/A
Please detail **housing and husbandry conditions**.	Yes	Described in Material and Methods

Plants and microbes	Information included in the manuscript?	In which section is the information available? (Reagents and Tools Table, Materials and Methods, Figures, Data Availability Section)
**Plants:** provide species and strain, ecotype and cultivar where relevant, unique accession number if available, and source (including location for collected wild specimens).	Not Applicable	
**Microbes:** provide species and strain, unique accession number if available, and source.	Yes	The use of bacteria (and RRID) for generating Crispr-cas9 constructs is described in methods

Human research participants	Information included in the manuscript?	In which section is the information available? (Reagents and Tools Table, Materials and Methods, Figures, Data Availability Section)
If collected and within the bounds of privacy constraints report on age, sex and gender or ethnicity for all study participants.	Yes	Reported in Supplementary Table 1

Core facilities	Information included in the manuscript?	In which section is the information available? (Reagents and Tools Table, Materials and Methods, Figures, Data Availability Section)
If your work benefited from core facilities, was their service mentioned in the acknowledgments section?	Yes	This is in acknowledgments

Design

Study protocol	Information included in the manuscript?	In which section is the information available? (Reagents and Tools Table, Materials and Methods, Figures, Data Availability Section)
If study protocol has been **pre-registered, provide DOI in the manuscript**. For clinical trials, provide the trial registration number **OR** cite DOI.	Not Applicable	
Report the **clinical trial registration number** (at ClinicalTrials.gov or equivalent), where applicable.	Not Applicable	

Laboratory protocol	Information included in the manuscript?	In which section is the information available? (Reagents and Tools Table, Materials and Methods, Figures, Data Availability Section)
Provide DOI OR other citation details if **external detailed step-by-step protocols** are available.	Not Applicable	

Experimental study design and statistics	Information included in the manuscript?	In which section is the information available? (Reagents and Tools Table, Materials and Methods, Figures, Data Availability Section)
Include a statement about **sample size** estimate even if no statistical methods were used.	Yes	In Material and Methods
Were any steps taken to minimize the effects of subjective bias when allocating animals/samples to treatment (e.g. **randomization procedure**)? If yes, have they been described?	Yes	Random cages were used for each groups
Include a statement about **blinding** even if no blinding was done.	Yes	
Describe **inclusion/exclusion criteria** if samples or animals were excluded from the analysis. Were the criteria pre-established?<br><br>If sample or data points were omitted from analysis, report if this was due to attrition or intentional exclusion and provide justification.	Not Applicable	
For every figure, are **statistical tests** justified as appropriate? Do the data meet the assumptions of the tests (e.g., normal distribution)? Describe any methods used to assess it. Is there an estimate of variation within each group of data? Is the variance similar between the groups that are being statistically compared?	Yes	This is described in data. Please note that due to the relatively low number of patient samples used we did not estimate variance in groups

Sample definition and in-laboratory replication	Information included in the manuscript?	In which section is the information available? (Reagents and Tools Table, Materials and Methods, Figures, Data Availability Section)
In the figure legends: state number of times the experiment was **replicated** in laboratory.	Yes	In Figure legends and in table refferreing to what patients data is in each figure
In the figure legends: define whether data describe **technical or biological replicates**.	Yes	In Figure legends and in table refferreing to what patients data is in each figure

Ethics

Ethics	Information included in the manuscript?	In which section is the information available? (Reagents and Tools Table, Materials and Methods, Figures, Data Availability Section)
Studies involving **human participants**: State details of **authority granting ethics approval** (IRB or equivalent committee(s), provide reference number for approval.	Yes	This information is contained within Study Design section
Studies involving **human participants**: Include a statement confirming that **informed consent** was obtained from all subjects and that the experiments conformed to the principles set out in the WMA Declaration of Helsinki and the Department of Health and Human Services Belmont Report.	Yes	This information is contained within Study Design section
Studies involving **human participants:** For publication of **patient photos**, include a statement confirming that consent to publish was obtained.	Not Applicable	
Studies involving experimental **animals**: State details of **authority granting ethics approval** (IRB or equivalent committee(s), provide reference number for approval. Include a statement of compliance with ethical regulations.	Yes	Descibed in Material and Methods
Studies involving **specimen and field samples:** State if relevant **permits** obtained, provide details of authority approving study; if none were required, explain why.	Not Applicable	

Dual Use Research of Concern (DURC)	Information included in the manuscript?	In which section is the information available? (Reagents and Tools Table, Materials and Methods, Figures, Data Availability Section)
Could your study fall under dual use research restrictions? Please check biosecurity documents and list of **select agents and toxins** (CDC): https://www.selectagents.gov/sat/list.htm	Not Applicable	
If you used a select agent, is the security level of the lab appropriate and reported in the manuscript?	Not Applicable	
If a study is subject to dual use research of concern regulations, is the name of the **authority granting approval and reference number** for the regulatory approval provided in the manuscript?	Not Applicable	

Reporting

The MDAR framework recommends adoption of discipline-specific guidelines, established and endorsed through community initiatives. Journals have their own policy about requiring specific guidelines and recommendations to complement MDAR.

Adherence to community standards	Information included in the manuscript?	In which section is the information available? (Reagents and Tools Table, Materials and Methods, Figures, Data Availability Section)
State if relevant guidelines or checklists (e.g., **ICMJE, MIBBI, ARRIVE, PRISMA**) have been followed or provided.	Yes	ARRIVE guidelines were followed as described in Material and Methods
For **tumor marker prognostic studies,** we recommend that you follow the **REMARK** reporting guidelines (see link list at top right). See author guidelines, under 'Reporting Guidelines'. Please confirm you have followed these guidelines.	Not Applicable	
For **phase II and III randomized controlled trials**, please refer to the **CONSORT** flow diagram (see link list at top right) and submit the CONSORT checklist (see link list at top right) with your submission. See author guidelines, under 'Reporting Guidelines'. Please confirm you have submitted this list.	Not Applicable	

Data Availability

Data availability	Information included in the manuscript?	In which section is the information available? (Reagents and Tools Table, Materials and Methods, Figures, Data Availability Section)
Have **primary datasets** been deposited according to the journal's guidelines (see 'Data Deposition' section) and the respective accession numbers provided in the Data Availability Section?	Yes	The dataset produced in this study has been deposited to Gene Expression Omnibus GSE226887 (https://www.ncbi.nlm.nih.gov/geo/query/acc.cgi?acc=GSE226887).
Were **human clinical and genomic datasets** deposited in a public access-controlled repository in accordance to ethical obligations to the patients and to the applicable consent agreement?	Yes	Samples from patients who had given informed consent were anonymised with no identifing data associated with it
Are **computational models** that are central and integral to a study available without restrictions in a machine-readable form? Were the relevant accession numbers or links provided?	Not Applicable	
If publicly available data were reused, provide the respective **data citations in the reference list**.	Yes	Relevant accession numbers are given in text
